# Water limitation alters pea seed compositional traits and the culturable bacterial fraction recovered from the rhizosphere

**DOI:** 10.3389/fpls.2026.1932989

**Published:** 2026-10-12

**Authors:** Joana Machado, Bruno Nogueira-Santos, Marta Nunes da Silva, Gianuario Fortunato, Carla Sancho Santos, Marta W. Vasconcelos

**Affiliations:** Centro de Biotecnologia e Química Fina, Escola Superior de Biotecnologia da Universidade Católica Portuguesa, Porto, Portugal

**Keywords:** antioxidant capacity, culturable rhizosphere bacteria, legumes, *Pisum sativum*, seed nutritional quality

## Abstract

**Introduction:**

Drought is one of the major environmental constraints affecting grain legume production, yet its impact on seed compositional traits remains less understood than its effects on yield. This study evaluated the effects of water limitation on plant growth, seed nutritional and biochemical traits in *Pisum sativum* L. cv. Tom Thumb. Additionally, culturable bacteria were isolated from the pea rhizosphere to assess whether water limitation was associated with differences in the isolates recovered under the two watering regimes.

**Methods:**

Plants were grown under controlled conditions and subjected to well-watered control conditions (80% field capacity, FC) or water-limited conditions (40% FC) until maturity.

**Results:**

Water limitation reduced shoot, pod, seed and total aerial fresh weight, whereas no statistically detectable differences were observed in the corresponding dry weights, pod number, or seed number. Water-limited conditions altered seed phytochemical composition, increasing saponin and flavonoid contents by 95% and 14%, respectively, and decreasing total phenolic content by 11%. ABTS antioxidant activity increased, while DPPH activity showed a marginal decrease. Endpoint-specific comparisons also indicated lower seed nitrogen (N), with a corresponding reduction in N-derived estimated crude protein, and lower zinc (Zn) concentrations, while molybdenum (Mo) showed a marginal decrease. However, none of these nutritional or mineral responses remained supported after false discovery rate adjustment. Culture-dependent analysis of rhizosphere bacteria showed that reduced water availability had limited effects on culturable bacterial abundance, although different bacterial isolates were recovered under the two watering regimes.

**Conclusions:**

Overall, water limitation altered pea seed composition and was associated with differences in culturable rhizosphere bacterial isolates, even when dry reproductive biomass was not detectably reduced.

## 1 Introduction

Climate change is increasing the frequency and severity of drought events, posing a growing threat to crop production and global food security (Fernandes et al., 2022). Among environmental constraints, water limitation is one of the most important limitations to crop production, as it reduces photosynthetic activity, carbon assimilation, and assimilate translocation, ultimately leading to substantial yield losses (Machado et al., 2025). These impacts are particularly relevant for grain legumes, which are major sources of plant protein, dietary fibre, minerals, and bioactive compounds, and play a key role in the transition towards more sustainable food systems.

Pea (*Pisum sativum* L.) is one of the most widely cultivated cool-season grain legumes and is increasingly used in traditional foods and plant-based products due to its high protein content and favourable nutritional profile. Pea also contributes to agricultural sustainability as a legume crop that can support lower-input production systems and improve soil fertility (Machado et al., 2025). However, pea is particularly sensitive to water deficit, especially during flowering and seed filling, when this limitation can impair pod development, seed growth, and the accumulation of storage reserves (Sehgal et al., 2018). Seed development is a critical stage involving the mobilisation of nutrients and the synthesis of storage compounds that ultimately determine the nutritional value of the harvested grain, including its protein, mineral, and bioactive compound composition (Sripathy and Groot, 2023). Water deficit during seed filling may therefore affect seed nutritional quality and influence the overall value of the harvested grain.

Although the effects of drought on plant growth and yield have been extensively investigated, less is known about how water limitation modifies seed nutritional quality, particularly when protein, mineral composition, and phytochemical traits are considered together. Evidence from lentil, chickpea, common bean, and soybean indicates that water deficit can reduce seed protein and essential amino acid contents while altering mineral accumulation, particularly that of iron (Fe), zinc (Zn), and phosphorus (P) (Sehgal et al., 2018). Water limitation can also influence secondary metabolism, resulting in changes in phytochemicals such as flavonoids, phenolic compounds, and saponins, which contribute to antioxidant activity and other health-promoting properties (Geraldo et al., 2022; Duarte et al., 2024; Machado et al., 2025). However, these responses are often species- and stress-dependent and remain insufficiently characterised in pea.

Beyond its direct effect on plant physiology, lower water availability can alter rhizosphere conditions by affecting soil water availability, nutrient mobility, and root exudation patterns (Wang et al., 2026). These changes may influence plant-associated bacterial populations, including bacterial taxa involved in stress-related plant-microbe interactions and drought-associated microbiome responses (Ali et al., 2022; Liu et al., 2025). In turn, changes in plant-associated bacterial populations may affect plant nutrition and stress response (Ali et al., 2022), yet culture-dependent rhizosphere bacterial responses are rarely examined alongside seed nutritional traits. Evaluating seed quality and rhizosphere bacterial responses within the same experimental framework may therefore provide a broader understanding of how pea plants respond to water deficit.

Accordingly, the present study evaluated the effects of water limitation at 40% field capacity (FC) on growth, selected plant-level traits, seed nutritional traits, bioactive compounds, and culturable rhizosphere bacterial isolates recovered under contrasting water regimes. By integrating plant, seed, and culture-dependent rhizosphere-related responses, this work provides insight into how water limitation affects seed quality and the culturable bacterial isolates recovered from the pea rhizosphere.

## 2 Materials and methods

### 2.1 Plant material and growth conditions

The experiment was conducted in an Aralab 10000 EHHF growth chamber under controlled conditions: 21 °C/16 °C day/night temperature, 70% relative humidity, photosynthetic photon flux density (PPFD) of 150 μmol m^-2^ s^-1^ and a 16 h photoperiod. Light was supplied by white LED lamps. PPFD was set at plant level and checked across representative pot positions before the start of the experiment using an LI-250 light meter equipped with a PAR sensor (LI-COR Biosciences, Lincoln, NE, USA), and pots were rotated periodically to minimise positional effects within the chamber. CO_2_ concentration was not independently controlled and therefore corresponded to ambient chamber conditions. Plants were arranged according to a completely randomised design and harvested at full maturity, 50 days after sowing.

Seeds of *Pisum sativum* cv. Tom Thumb were sown into single-plant pots (15 cm high, 12 cm diameter). Tom Thumb is a compact/dwarf garden pea cultivar, suitable for controlled-environment pot experiments because of its small stature and short growth cycle. Pots were filled with 200 g of a Siro^®^ Universal commercial substrate (SIRO Portugal), thoroughly homogenised with perlite at a 3:1 ratio before pot filling. The substrate was used as supplied by the manufacturer and was not sterilised. No rhizobial inoculation, additional fertiliser, liming material, or other nutrient amendment was applied during the experiment.

Field capacity (FC) was operationally defined for the substrate-perlite mixture used in this experiment using a gravimetric approach. Pots containing the substrate-perlite mixture were saturated with water until drainage occurred and then allowed to drain until free drainage ceased. The resulting pot weight was used as the reference corresponding to 100% FC. Target pot weights corresponding to 90%, 80%, and 40% FC were then calculated from this reference and used to adjust daily irrigation gravimetrically. Plants were initially watered to 100% FC. Until seedling establishment, corresponding to first node appearance, pots were maintained at 90% FC by adding water as needed. After seedling establishment, pots were covered to minimise evaporation and plants were assigned to two watering regimes: well-watered control plants maintained at 80% FC and water-limited plants maintained at 40% FC until full maturity (50 days after sowing).

Target substrate water content was maintained by weighing all pots individually and watering them daily throughout the experiment. The amount of water added to each pot was calculated from the difference between the target pot weight for each watering regime and the actual pot weight recorded on that day. No drainage was observed during the experiment, and evaporation was minimised by covering the pots. To account for the increasing contribution of plant fresh weight to total pot weight during plant development, three additional pots per treatment were destructively sampled each week. The mean plant fresh weight obtained from these destructive samples was used to correct substrate water-content estimates when adjusting irrigation.

At harvest, 50 days after sowing, seeds used for biochemical and nutritional analyses were collected from visually mature pods. Visibly immature, malformed, or damaged seeds were excluded. Growth, physiological, seed biochemical, seed nutritional, and culturable bacterial abundance measurements were performed using independent biological replicates, with endpoint-specific sample sizes reported in **Supplementary Table S1**. For seed biochemical and nutritional analyses, five independent biological replicates were used per treatment, each consisting of all mature seeds available from one individual plant. Seeds from different plants were not pooled. The seed material from each plant was immediately frozen in liquid nitrogen, stored at −80 °C, lyophilised, ground, and homogenised separately. For each analytical assay, the required sample mass was weighed from the homogenised material corresponding to that individual plant. Rhizosphere soil samples were collected from individual plants by gently removing loosely attached soil and collecting the soil tightly adhering to the root surface. These samples were collected into sterile containers and processed immediately after sampling. Sample collection and preparation of the rhizosphere suspensions occurred in the same day, and all samples were handled using the same procedure.

### 2.2 Plant growth and physiological parameters

Shoot, pod and seed fresh weight (FW) were recorded separately at harvest. Shoot biomass corresponded to stems and leaves and excluded pods and seeds. Dry weights (DW) used for gravimetric biomass determination were obtained after oven-drying at 105 °C for 48 h. Pod number and seed number, as well as plant height, were recorded. Total aerial biomass was calculated as the sum of shoot, pod, and seed biomass. Leaf chlorophyll content was measured on the youngest fully expanded leaf using the non-destructive chlorophyll meter SPAD-502 (Konica Minolta, Tokyo, Japan). Cumulative water supplied was calculated for each pot as the sum of all daily water additions over the experimental period. Aerial dry biomass produced per unit of cumulative water supplied was calculated as total aerial dry weight divided by cumulative water supplied.

### 2.3 Antioxidant activity and bioactive compounds

For all the following analyses, seeds were lyophilised and ground using a Retsch Cyclone Mill and stored at -20 °C until use.

#### 2.3.1 Extraction of bioactive compounds

For antioxidant activity and bioactive compounds analyses, 150 mg of lyophilised and ground seed material was extracted with 1 mL of 80% methanol. Samples were sonicated in an ultrasonic bath (Branson 3510, Connecticut, USA) at 42 kHz for 30 min at room temperature, followed by centrifugation at 3000 x g for 20 min. The supernatants were collected and stored in the dark at -20 °C until analysis.

#### 2.3.2 Antioxidant activity

Antioxidant activity was assessed using the ABTS [2,2-azinobis(3-ethylbenzothiazoline-6-sulfonic acid)] and DPPH (2,2-diphenyl-1-picrylhydrazyl) radical scavenging assays, using Trolox (25–175 μM) as the standard.

The ABTS assay was performed in a 96-well microplate following Gonçalves et al. (2009) with slight modifications. The ABTS working solution was adjusted to an absorbance of 0.700 ± 0.020 at 734 nm. Subsequently, 180 μL of ABTS working solution was mixed with 20 μL of sample extract, Trolox standard, or methanol as the reagent blank. After incubation for 5 min at 30 °C, absorbance was measured at 734 nm using a Multidetection microplate reader (Synergy H1, Vermont, USA). The decrease in absorbance relative to the reagent blank was used for quantification. Trolox was used as the calibration standard over the range of 0.05–0.35 μmol mL^-^¹, and the calibration curve showed a coefficient of determination of 0.9979.

The DPPH assay was carried out in a 96-well microplate according to Brand-Williams et al. (1995) with modifications. The DPPH working solution was adjusted to an absorbance of 0.600 ± 0.100 at 517 nm. Subsequently, 175 μL of DPPH working solution was mixed with 25 μL of sample extract, Trolox standard, or methanol as the reagent blank. After incubation for 30 min at 25 °C in the dark, absorbance was measured at 517 nm using a Multidetection microplate reader (Synergy H1, Vermont, USA). The decrease in absorbance relative to the reagent blank was used for quantification. Trolox was used as the calibration standard over the range of 0.025–0.250 µmol mL^-^¹, and the calibration curve showed a coefficient of determination of 0.9980.

Results from both assays were expressed as µmol trolox equivalents (TE) g^-1^ DW. Measurements were performed in technical triplicate for each of the five independent biological replicates per treatment and averaged before statistical analysis.

#### 2.3.3 Phenolics and flavonoids quantification

Total phenolic content (TPC) was determined using the Folin–Ciocalteu reagent as described by Machado et al. (2023) with slight modifications. Briefly, 20 μL of extract, methanol or gallic acid standard was mixed with 100 μL of Folin–Ciocalteu reagent 1:10 (v/v) and 100 μL of 7.5% (w/v) sodium carbonate in a 96-well plate. After 30 min of incubation at room temperature in the dark, absorbance was measured at 750 nm using a Multidetection microplate reader (Synergy H1, Vermont, USA). Gallic acid was used as the calibration standard over the range of 0.010–0.125 mg mL^-^¹, and the calibration curve showed a coefficient of determination of 0.9922. Results were expressed as mg gallic acid equivalents (GAE) g^-1^ DW. Measurements were performed in technical triplicate for each of the five independent biological replicates per treatment and averaged before statistical analysis.

Total flavonoid content (TFC) was determined following Zhang et al. (2015), with slight modifications. Briefly, 62.5 μL of extract, methanol or catechin standard was mixed with 275 μL of sodium nitrite (0.066 M), vortexed, and incubated for 5 min at room temperature. Subsequently, 37.5 μL of aluminium chloride (0.75 M) was added, followed by vortexing and incubation for 6 min at room temperature. Afterwards, 250 μL of sodium hydroxide (0.5 M) was added, and the mixture was vortexed again. An aliquot of 200 μL of the final reaction mixture was transferred to a 96-well microplate, and absorbance was measured at 510 nm using a Multidetection microplate reader (Synergy H1, Vermont, USA). Catechin was used as the calibration standard over the range of 0.005-0.250 mg mL^-1^), and the calibration curve showed a coefficient of determination of 0.9978. Results were expressed as mg catechin equivalents (CE) g^-1^. Measurements were performed in technical triplicate for each of the five independent biological replicates per treatment and averaged before statistical analysis.

#### 2.3.4 Phytic acid quantification

Phytic acid was quantified using the K-PHYT enzymatic assay kit (Megazyme Inc, Bray, Ireland) according to the manufacturer’s instructions, with all assay volumes proportionally reduced for adaptation to a 96-well microplate format. Approximately 250 mg of ground, lyophilised seed material from each biological replicate was extracted in 5 mL of hydrochloric acid (0.66 M). Total and free phosphorus were determined enzymatically, and phytic acid content was calculated from the difference between the two determinations using the conversion procedure specified by the manufacturer. Absorbance was measured at 655 nm using a Multidetection microplate reader (Synergy H1, Vermont, USA). The phosphorus calibration covered 0–7.5 µg per assay and showed a coefficient of determination greater than 0.9999. The oat control supplied with the kit was analysed alongside the samples. Measurements were performed in technical triplicate for each of the five independent biological replicates per treatment and averaged before statistical analysis. Results were expressed as mg phytic acid g^-1^ dry weight.

#### 2.3.5 Total Saponins quantification

Total saponin content was quantified using the nonspecific vanillin–sulphuric acid colorimetric assay, according to Hiai et al. (1976), with slight modifications. Approximately 80 mg of ground, lyophilised seed material from each biological replicate was extracted in 2 mL of ethanol. Briefly, 100 μL of extract, ethanol blank, or diosgenin standard was mixed with 100 μL of 8% (w/v) vanillin and 1 mL of 72% (v/v) sulphuric acid. Samples were incubated for 15 min at 60 °C with vigorous shaking and then cooled on ice. After cooling, 200 μL of the reaction mixture were transferred to a 96-well microplate. Absorbance was measured at 560 nm using a Multidetection microplate reader (Synergy H1, Vermont, USA). Diosgenin was used as the calibration standard over the range of 0.015–0.150 mg mL^-^¹, and the calibration curve showed a coefficient of determination of 0.9938. Results were expressed as mg diosgenin equivalents (DE) g^-1^ DW. Measurements were performed in technical triplicate for each of the five independent biological replicates per treatment and averaged before statistical analysis.

#### 2.3.6 Total nitrogen, estimated crude protein and mineral quantification

Seed total N concentration was determined using the Dumas combustion method. Approximately 100 mg of ground, lyophilised seed material from each biological replicate was weighed into an aluminium crucible and analysed using a DumatecTM 8000/FOSS nitrogen analyser. The combustion and reduction reactors were operated at 979 and 650 °C, respectively, with helium flow rates of 195.0 and 200 mL min^−1^, an oxygen flow rate of approximately 300 mL min^−1^ and a pressure of approximately 1200 mBar. Ethylenediaminetetraacetic acid was used as the calibration standard, and analytical blanks were included to monitor background N. Estimated crude protein content was calculated directly from total seed N using the conventional N-to-protein conversion factor of 6.25, which was retained to facilitate comparison with studies reporting crude protein estimates (Mossé, 1990; Mariotti et al., 2008).

Macro- and micromineral concentrations were determined by inductively coupled plasma optical emission spectrometry (ICP-OES) according to Machado et al. (2025). Briefly, 250 mg of the ground lyophilised seed material from each biological replicate was mixed with 10 mL of 65% nitric acid in a Teflon reaction vessel (MARSXpress, CEM Corporation) and subjected to microwave-assisted digestion using a MARS 6 Microwave Digestion System (CEM Corporation, USA). The digestion procedure consisted of a 15 min ramp to 175 °C, followed by a 15 min hold at 175 °C, with a pressure of 800 psi and a power of 900 W. After the digestion, the clear solution was brought to 50 mL with ultrapure water. Mineral concentrations were then determined using an Optima 7000 DV ICP-OES system (PerkinElmer, USA) with a radial configuration. External multipoint calibration curves were prepared for each quantified element, covering the concentration ranges of the analysed samples, with coefficients of determination ranging from 0.9986 to 0.9998. The analytical wavelengths were 203.845 nm for Mo, 213.857 nm for Zn, 214.914 nm for P, 257.610 nm for Mn, 259.939 nm for Fe, 279.077 nm for Mg, 317.933 nm for Ca, and 769.896 nm for K. Digestion blanks and NIST Standard Reference Material SRM 1573a Tomato Leaves were processed alongside the seed samples for analytical quality control. The measured concentrations of the certified elements were within the corresponding certified ranges. Each analytical solution was measured using three instrumental readings, and blank-corrected intensities were used for quantification.

### 2.4 Isolation and enumeration of culturable rhizosphere bacteria

Rhizosphere samples were suspended in sterile 0.85% NaCl solution at a 1:10 ratio (w/v) and incubated for 1 h at room temperature under agitation to detach microorganisms from soil particles. The resulting suspension was centrifuged at 5,000 × g for 5 min. The undiluted supernatant (10^0^) and serial dilutions from 10^-1^ to 10^-^³ were prepared in sterile 0.85% NaCl solution. For the undiluted supernatant and each serial dilution, 100 μL aliquots were plated in technical triplicate on four culture media with different nutritional compositions: Luria-Bertani agar (LB), Nutrient Agar No. 1 (NA), Yeast Extract Agar (YEA), and Tryptic Soy Agar (TSA). Plates were incubated at 27 °C for 72 h. Plates containing 10–100 colonies were used for enumeration. Colony-forming units (CFU) were calculated as:


$$\begin{array}{l} {\text{CFU mL}}^{-1}=\text{mean colony count}\\ \times \text{dilution factor}/\text{plated volume }(\text{mL}). \end{array}$$

When no colonies were detected at a given dilution, enumeration was based on the next less-diluted plate with detectable colonies, when available. The theoretical lower detection limit was 10 CFU mL^-^¹, corresponding to one detectable colony in 100 μL of the undiluted supernatant. Technical replicate plates were averaged within each independent rhizosphere sample before statistical analysis. Three independent rhizosphere samples were analysed per treatment (*n* = 3). Representative colonies encompassing the visually distinct morphotypes observed on each medium were selected based on colony size, pigmentation, shape, margin, texture, and opacity. Colony selection was guided by standard descriptions of bacterial colony morphology and culture characteristics reported in commonly used microbiological reference manuals (Holt et al., 1994; Zimbro et al., 2009; Goodfellow et al., 2012). These morphological criteria were used only for the selection of visually distinct colonies for isolation and were not used for taxonomic identification. Selected colonies were purified by successive streaking until pure cultures were obtained.

### 2.5 Molecular identification and phylogenetic analysis of bacterial isolates

Representative colonies were selected from each culture medium based on colony morphology and purified by repeated streaking. Genomic DNA was extracted from purified bacterial colonies by suspending a single colony in 200 μL of nuclease-free water, followed by incubation at 96 °C for 5 min. Samples were immediately cooled on ice and centrifuged at 5,000 × g for 5 min. The resulting supernatant was transferred to a new microtube and used as a template for downstream molecular analyses. DNA concentration was determined using a NanoPhotometer™ UV/Vis spectrophotometer (Implen GmbH, Germany).

Nearly full-length 16S rRNA genes were amplified using primers 27F (5′-GAGTTTGATCCTGGCTCA-3′) and 1492R (5′-TACCTTGTTACGACTT-3′) with NZYTaq II 2× Green Master Mix (NZYTech, Portugal). PCR amplifications were performed in a DOPPIO thermocycler (VWR, USA) with an initial denaturation at 95 °C for 5 min, followed by 25 cycles of 95 °C for 30 s, 54 °C for 30 s and 72 °C for 1 min, and a final extension step at 72 °C for 5 min. Amplified products were verified by electrophoresis on 1.5% agarose gels prepared in TAE buffer and stained with SYBR^®^ Safe DNA Gel Stain (Invitrogen, UK).

A total of 84 purified bacterial isolates were selected for Sanger sequencing by STAB VIDA (Lisbon, Portugal). The obtained 16S rRNA gene sequences were compared against the NCBI nucleotide database using BLASTn for preliminary taxonomic affiliation. BLAST results were manually curated based on percentage identity, query coverage, E value, alignment quality and taxonomic consistency among the top BLASTn hits. Reference sequences were considered suitable for downstream phylogenetic analysis when they provided a sufficiently strong and informative match to the corresponding isolate, with high sequence identity and broad query coverage. Matches showing substantially reduced sequence identity, low query coverage, weak or ambiguous alignments, or corresponding to uncultured/poorly characterised sequences were excluded. Of these, 27 isolates were excluded before analysis due to poor sequencing quality or mixed chromatograms indicative of co-isolation, resulting in 57 high-quality sequences for further taxonomic evaluation. From the remaining matches, 11 showing an identity below 95%, a query coverage below 70%, or corresponding to uncultured/environmental clones were excluded from phylogenetic reconstruction (**Supplementary Table S3**). The complete identification dataset, including BLAST metrics, GenBank accessions, isolation conditions, culture media, dilution levels, colony morphology, and exclusion criteria, is provided in **Supplementary Table S3**.

The final curated dataset included only isolate sequences (46) and reference 16S rRNA sequences providing adequate sequence similarity and coverage for phylogenetic comparison. 16S rRNA locus gene sequences were aligned using DECIPHER version 3.6.0 (Wright, 2016) and converted into a phyDat object using the phangorn package (Schliep, 2011). An unrooted neighbour-joining tree was initially constructed from maximum-likelihood sequence distances and used as the starting tree for maximum-likelihood inference. Candidate nucleotide substitution models were evaluated using the modelTest function in phangorn, and the best-fitting model was selected based on the Akaike Information Criterion (AIC). Maximum Likelihood phylogenetic reconstruction was conducted using phangorn implementing the General Time Reversible (GTR) substitution model, incorporating a gamma-distributed rate heterogeneity across sites (+G) and a proportion of invariable sites (+I). Branch support was assessed using 1,000 non-parametric bootstrap replicates, and bootstrap support values were displayed on internal nodes of the phylogenetic tree. The tree was visualised using ggtree in a circular layout, with isolation condition (well-watered or water-limited) incorporated as sample metadata. Specific clades were highlighted to indicate closely related bacterial isolates recovered exclusively under control or water-limited conditions.

### 2.6 Statistical analysis

Statistical analyses of plant physiological and biochemical parameters were performed using GraphPad Prism version 10.3.1 for macOS (GraphPad Software, Boston, MA, USA; www.graphpad.com). Biological replicates were used as the experimental units, and technical replicates, when present, were averaged before statistical analysis. Normality was assessed using the Shapiro-Wilk and Kolmogorov-Smirnov tests. For variables meeting the normality assumption, differences between well-watered or water-limited plants were analysed using Welch’s unpaired t-test, without assuming equal standard deviations. Variables that did not meet the normality assumption, namely pod number, seed number, seed dry weight, seed Mn concentration, and seed ABTS activity, were analysed using the Mann-Whitney test.

Data are expressed as mean ± standard deviation (SD). Endpoint-specific sample size, exact p-values, FDR-adjusted q-values, treatment effect estimates, confidence intervals, and the statistical test used for each endpoint are provided in **Supplementary Table S1**. Significance levels are denoted as follows: * *p* < 0.05; ** *p* < 0.01; *** *p* < 0.001; **** *p* < 0.0001; n.s., non-significant. As a sensitivity analysis, false discovery rate (FDR) adjustment was performed within families of related quantitative endpoints using the two-stage step-up method of Benjamini, Krieger and Yekutieli, with Q set to 5% (Benjamini et al., 2006). Because estimated crude protein was calculated directly from seed N, it was not analysed or included in the FDR sensitivity analysis as an independent endpoint. Its group means, SDs, treatment effect estimate, and confidence interval are deterministic transformations of the corresponding seed N values.

Analyses and visualisations of rhizosphere bacterial data were performed in R version 4.5.0 (R Core Team, 2025). Bar plots showing mean ± SD were generated using the ggplot2 version 4.0.3 (Wickham, 2016). The circular phylogenetic tree and its mapped external metadata rings, including isolation conditions and taxonomic clades, were generated using the ggtree (Yu et al., 2017) and ggtreeExtra (Xu et al., 2021) packages.

Culturable bacterial abundance was expressed as CFU mL^-1^ and log_10_-transformed [log_10_(x+1)] prior to statistical analysis. Each independent rhizosphere sample was considered the experimental unit, with three independent biological replicates per treatment. Differences between well-watered or water-limited treatments were assessed separately within each culture medium using Welch’s two-sample t-tests. Each medium was analysed separately because the media represent distinct selective growth conditions and therefore provide medium-specific measures of culturable bacterial recovery rather than repeated measurements of the same bacterial population. Resulting *p*-values were adjusted across culture media using the Benjamini–Hochberg FDR procedure (Benjamini and Hochberg, 1995). Statistical significance was defined as adjusted *p* < 0.05.

## 3 Results

### 3.1 Plant growth and physiological parameters

Growth- and physiology-related parameters were measured to evaluate the effects of water limitation on *P. sativum* cv. Tom Thumb. Water limitation at 40% FC significantly decreased the fresh weights of shoots (by 14%), pods (by 12%), seeds (by 8%) and aerial part (by 13%) **Supplementary Figure S1**, **Supplementary Table S1**). These fresh-weight responses remained supported after FDR adjustment within the growth, physiological, and water-related endpoint family (**Supplementary Table S1**). In contrast, no statistically detectable differences were observed for the corresponding dry weights (**Figure 1**).

**Figure 1:**
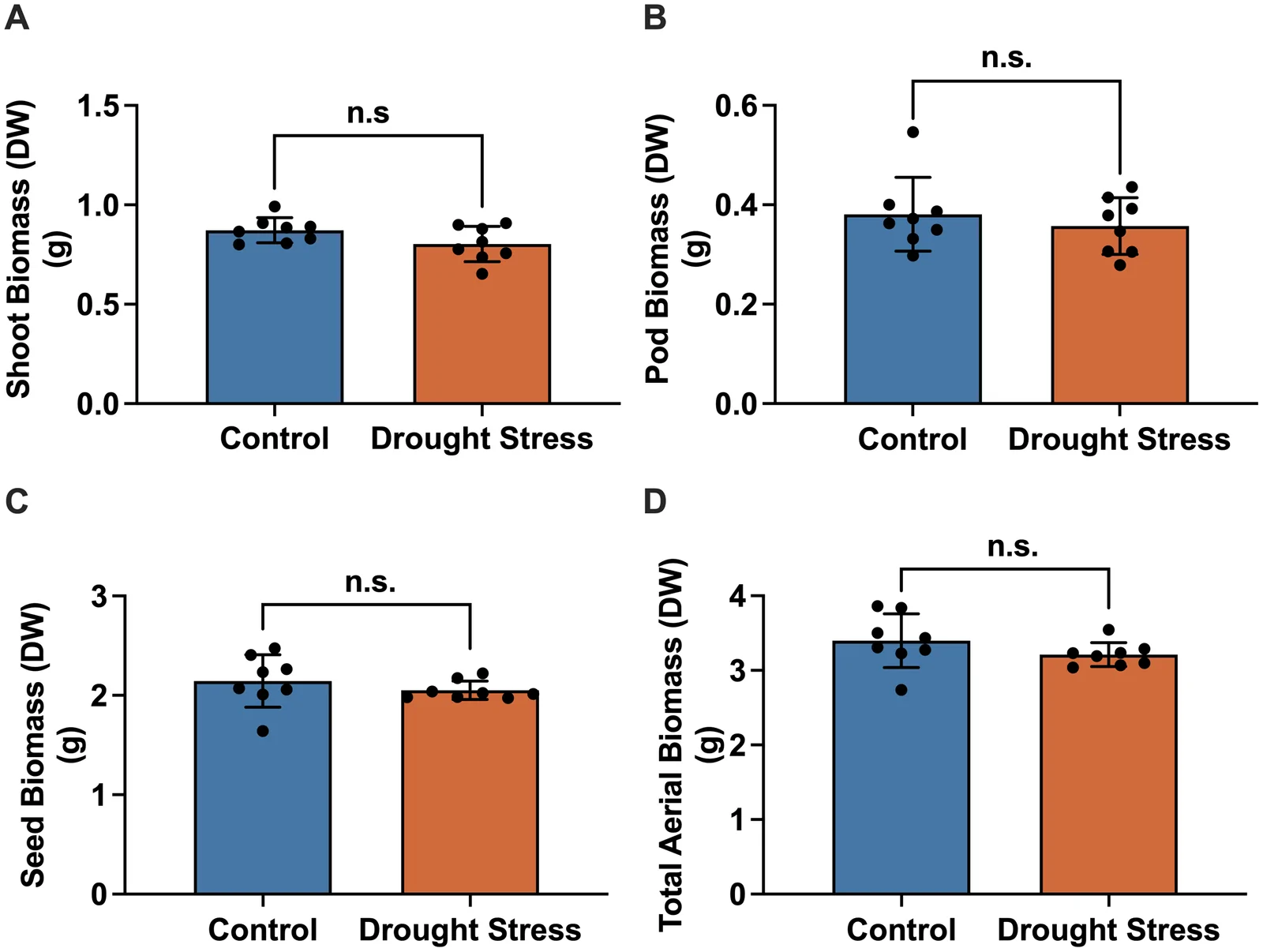
Shoot biomass **(A)**, pod biomass **(B)**, seed biomass **(C)**, and total aerial biomass **(D)**, expressed as dry weight of *Pisum sativum* cv. Tom Thumb plants subjected to well-watered control conditions (80% FC) or water-limited condition (40% FC). Individual points represent independent biological replicates. Bars and error bars indicate mean ± SD. n.s. denotes non-significant differences based on the endpoint-specific statistical test described in Section 2.6. Endpoint-specific sample sizes, exact p-values, FDR-adjusted q-values, treatment effect estimates, 95% confidence intervals, and statistical tests are provided in **Supplementary Table S1**.

No statistically detectable differences were observed in pod number or seed number between treatments, while plant height was significantly reduced by 11% under water-limited conditions (**Figure 2**, **Supplementary Table S1**).

**Figure 2:**
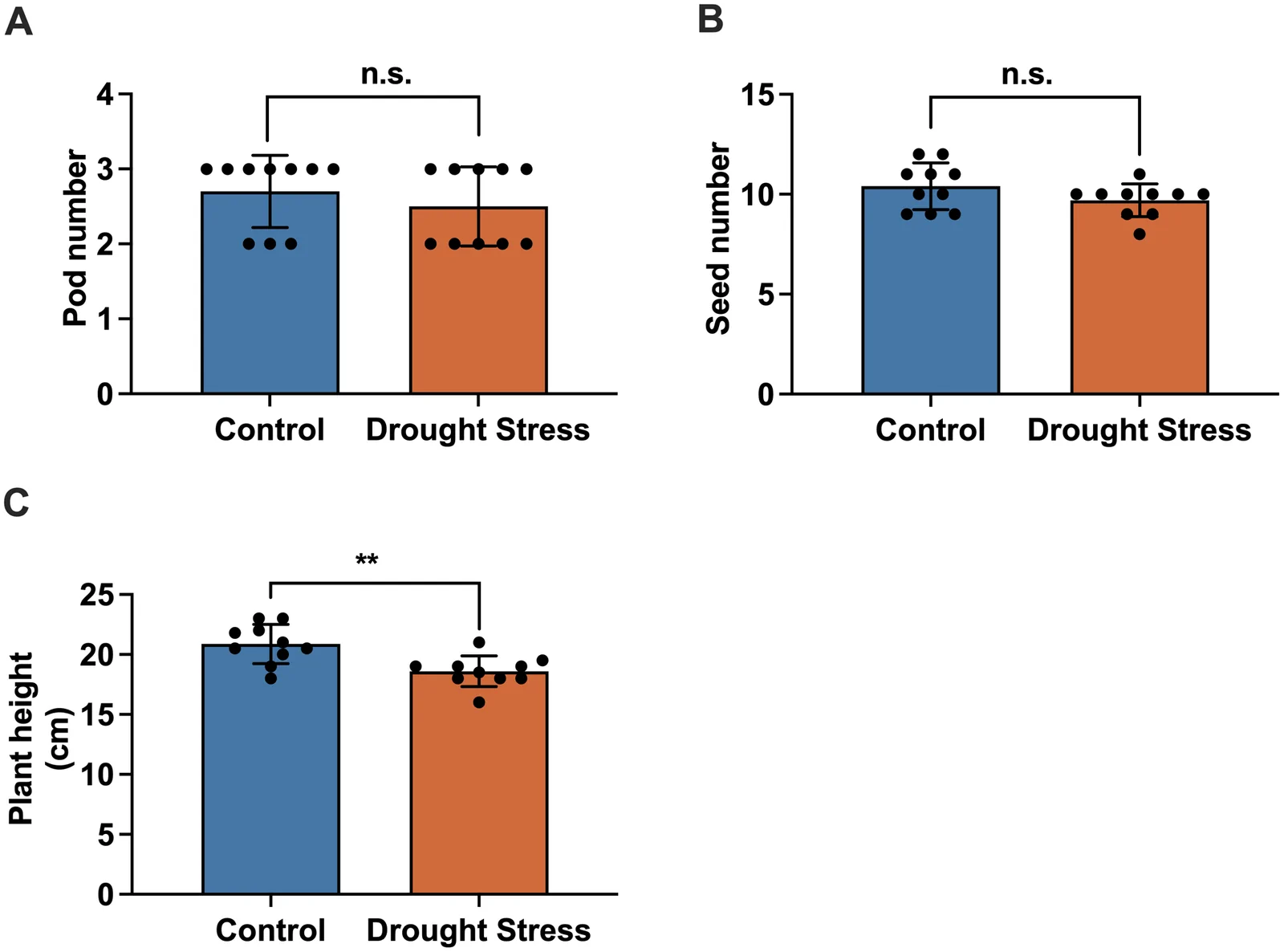
Pod number **(A)**, seed number **(B)**, and plant height **(C)** of *Pisum sativum* cv. Tom Thumb plants subjected to well-watered control conditions (80% FC) or water-limited condition (40% FC). Individual points represent independent biological replicates. Bars and error bars indicate mean ± SD. Asterisks indicate statistically significant differences based on the endpoint-specific statistical test described in Section 2.6, while n.s. denotes non-significant differences. Endpoint-specific sample sizes, exact p-values, FDR-adjusted q-values, treatment effect estimates, 95% confidence intervals, and statistical tests are provided in **Supplementary Table S1**.

Regarding physiological parameters, chlorophyll content, estimated by SPAD, did not differ statistically between treatments. Water-limited plants received 17% less cumulative water than control plants over the experimental period. When expressed relative to cumulative water supplied, aerial dry biomass was 12% higher under water limitation than under control conditions (**Figure 3**, **Supplementary Table S1**). Both cumulative water supplied and aerial dry biomass per unit of cumulative water supplied remained supported after FDR adjustment (**Supplementary Table S1**).

**Figure 3:**
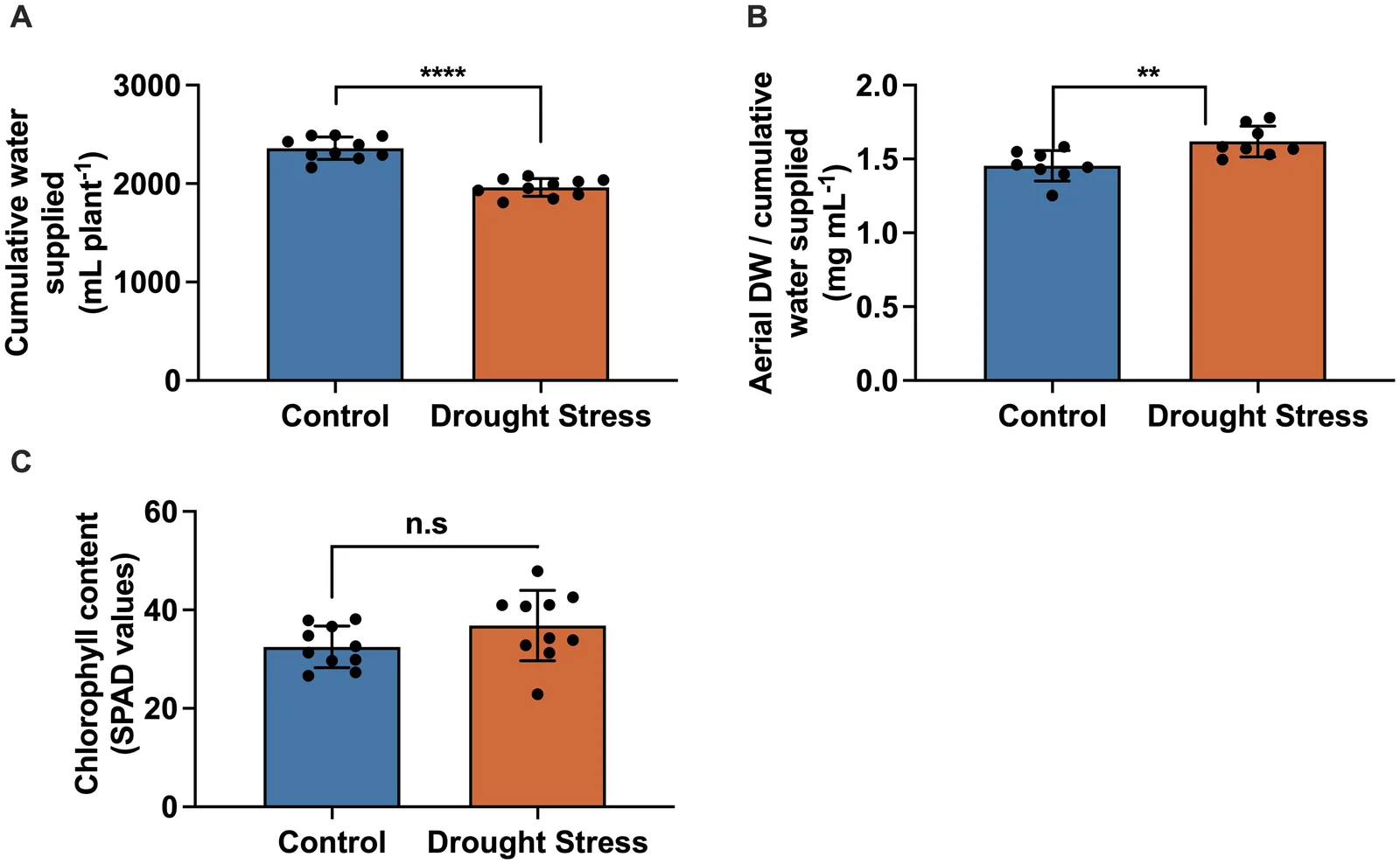
Cumulative water supplied **(A)**, aerial dry biomass produced per unit of cumulative water supplied **(B)**, and estimated chlorophyll content **(C)** of *Pisum sativum* cv. Tom Thumb plants subjected to well-watered control conditions (80% FC) or water-limited condition (40% FC). Individual points represent independent biological replicates. Bars and error bars indicate mean ± SD. Asterisks indicate statistically significant differences based on the endpoint-specific statistical test described in Section 2.6, while n.s. denotes non-significant differences. Endpoint-specific sample sizes, exact p-values, FDR-adjusted q-values, treatment effect estimates, 95% confidence intervals, and statistical tests are provided in **Supplementary Table S1**.

### 3.2 Antioxidant activity, bioactive compounds and nutritional traits

Water limitation significantly affected seed antioxidant activity and phytochemical composition, with assay-dependent responses (**Figure 4**, **Supplementary Table S1**). Compared with control plants, water-limited plants showed significantly higher ABTS antioxidant activity (11% increase), while DPPH activity showed a marginal decrease.

**Figure 4:**
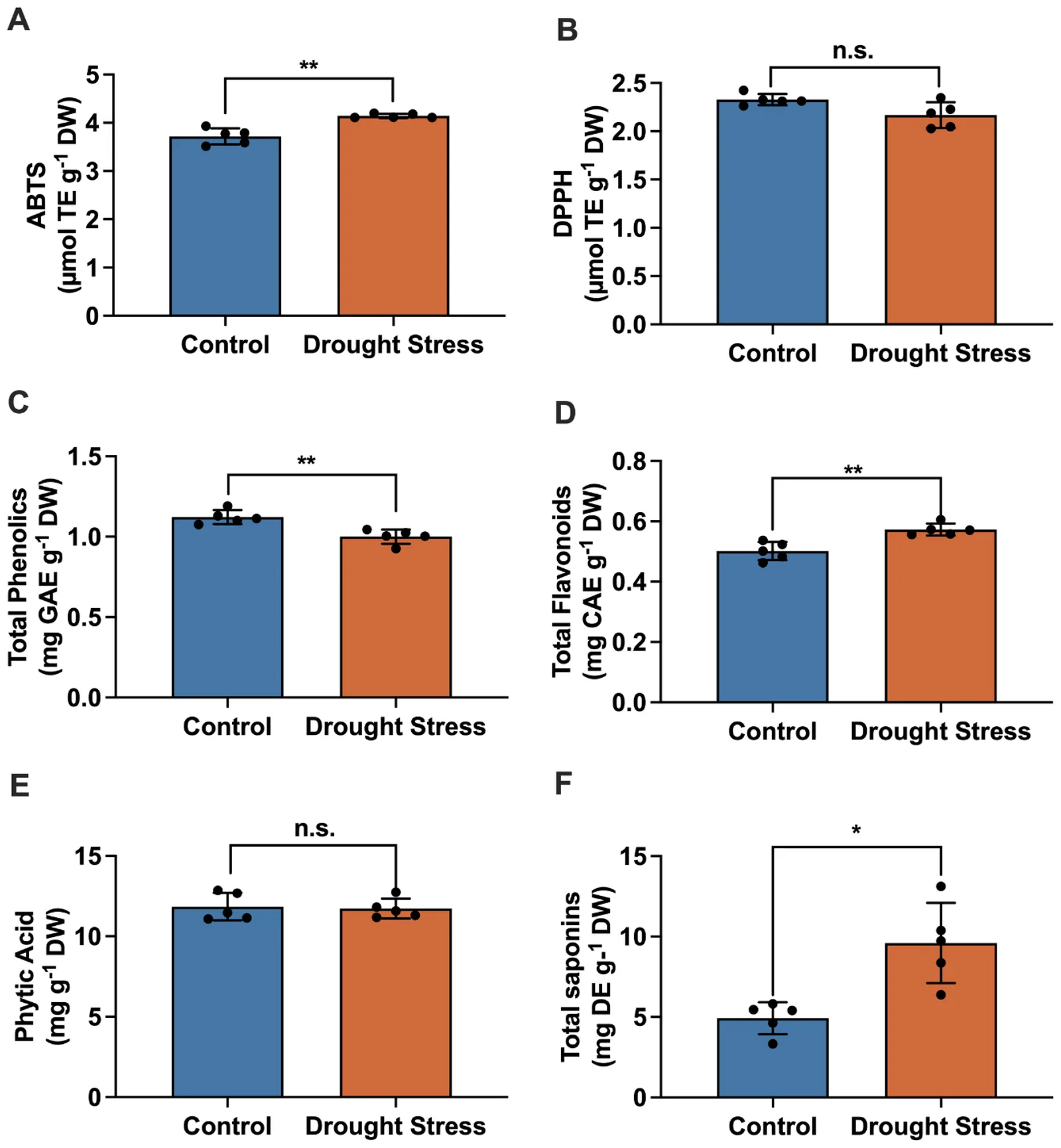
Seed biochemical traits of *Pisum sativum* cv. Tom Thumb plants subjected to well-watered control conditions (80% FC) or water-limited condition (40% FC): ABTS antioxidant activity **(A)**, DPPH antioxidant activity **(B)**, total phenolics **(C)**, total flavonoids **(D)**, phytic acid **(E)**, and total saponins **(F)**. Individual points represent independent biological replicates. Bars and error bars indicate mean ± SD. Asterisks indicate statistically significant differences based on the endpoint-specific statistical test described in Section 2.6, while n.s. denotes non-significant differences. Endpoint-specific sample sizes, exact p-values, FDR-adjusted q-values, treatment effect estimates, 95% confidence intervals, and statistical tests are provided in **Supplementary Table S1**.

Water-limited conditions also significantly altered the seed phytochemical profile. TFC and saponin content significantly increased by 14 and 95%, respectively, whereas TPC significantly decreased by 11% (**Figure 4**, **Supplementary Table S1**). These responses remained supported after FDR adjustment (**Supplementary Table S1**). No statistically detectable difference was observed for phytic acid (**Figure 4**, **Supplementary Table S1**).

Seed macro- and micromineral composition showed weaker responses to water limitation than phytochemical traits (**Figure 5**, **Supplementary Table S1**). Endpoint-specific comparisons indicated an 8% lower seed N concentration under water limiting conditions. Because estimated crude protein was calculated directly from seed N, the corresponding N-derived estimate was also 8% lower water limitation. Endpoint-specific comparisons also indicated a 7% lower Zn concentration, while Mo showed a marginal decrease. However, none of the mineral or nutritional endpoints remained supported after FDR adjustment within this endpoint family (**Supplementary Table S1****).** The concentrations of K, P, Ca, Mg, Fe, and Mn did not differ statistically between treatments.

**Figure 5:**
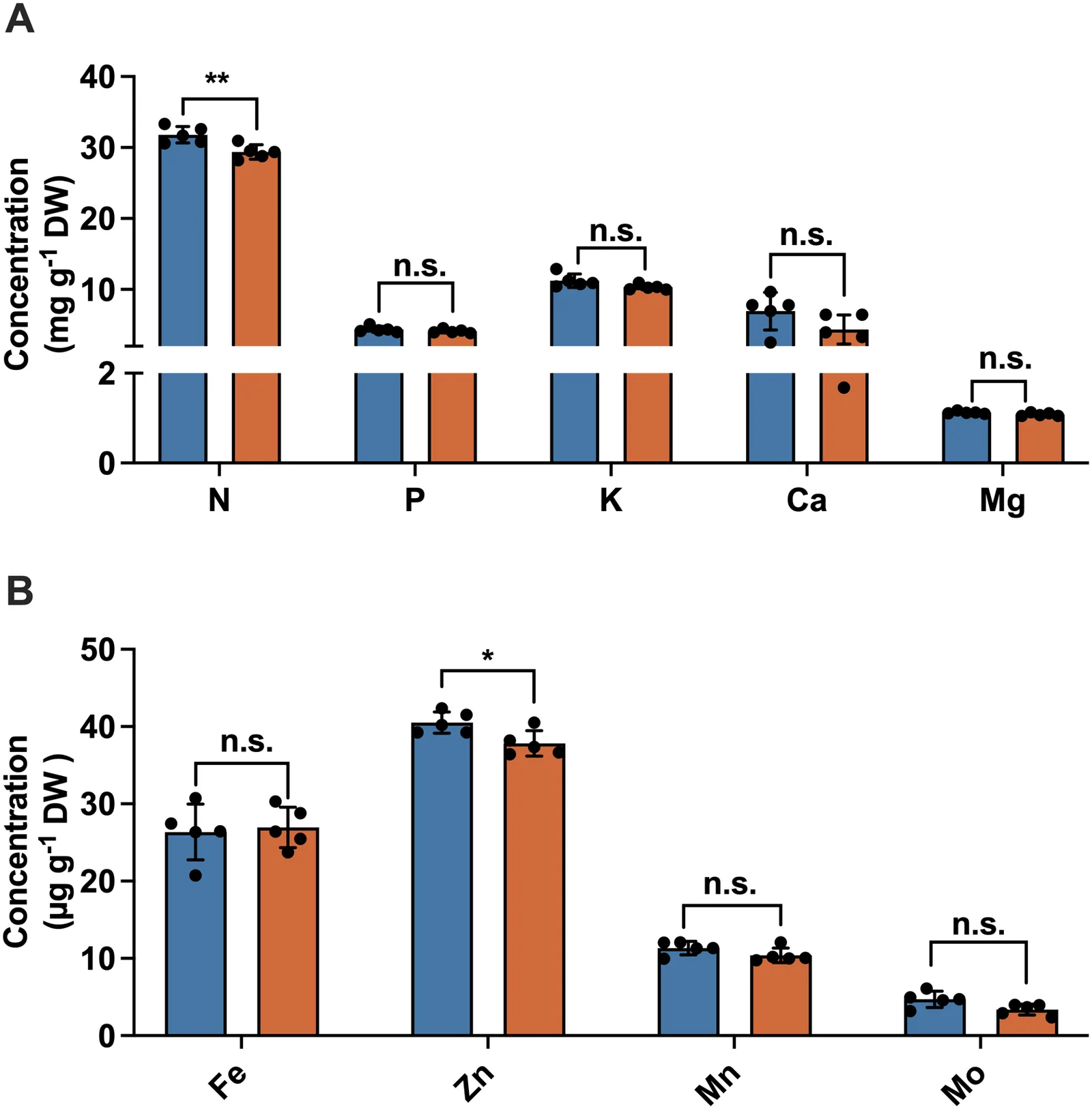
Seed macro- and micromineral composition of *Pisum sativum* cv. Tom Thumb plants subjected to well-watered control conditions (80% FC) and water-limited condition (40% FC). Panel **(A)** presents seed nitrogen (N), potassium (K), phosphorus (P), calcium (Ca), and magnesium (Mg) concentrations. Panel **(B)** presents seed iron (Fe), zinc (Zn), molybdenum (Mo), and manganese (Mn) concentrations. Individual points represent independent biological replicates. Bars and error bars indicate mean ± SD. Asterisks indicate statistically significant differences based on the endpoint-specific statistical test described in Section 2.6, while n.s. denotes non-significant differences. Endpoint-specific sample sizes, exact p-values, FDR-adjusted q-values, treatment effect estimates, 95% confidence intervals, and statistical tests are provided in **Supplementary Table S1**.

### 3.3 Culturable bacterial abundance and phylogenetic relationships of recovered rhizosphere isolates

Culturable bacteria were recovered from rhizosphere samples collected from control and water-limited plants using four culture media. Culturable bacterial abundance varied across culture media, with values ranging from approximately 10^3^ to 10^4^ CFU mL^-^¹ of rhizosphere suspension (**Figure 6B**).

**Figure 6:**
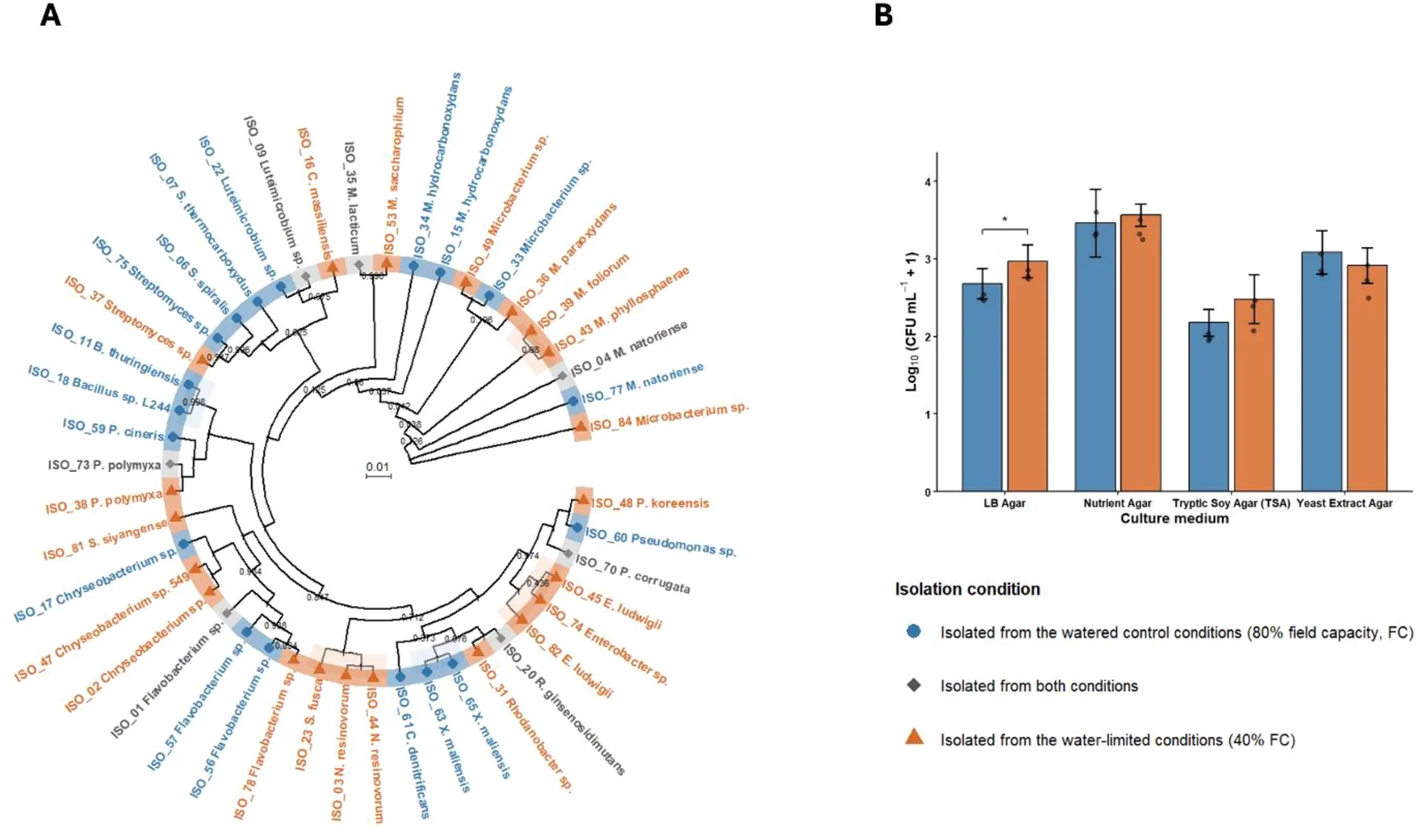
Phylogenetic relationships among culturable rhizosphere bacterial isolates recovered from rhizosphere samples under well-watered (control; 80% FC) or water-limited condition (40% FC) conditions, together with medium-specific culturable bacterial abundance. **(A)** Circular maximum-likelihood phylogenetic tree based on 16S rRNA gene sequences from the recovered study isolates and selected public reference sequences. Study isolates are identified by ISO labels, whereas reference sequences are labelled separately by taxon name and accession number. Isolates recovered exclusively under drought (orange), exclusively under control (blue), or under both conditions (grey) are shown. Here, *“Shared”* indicates taxonomic groups represented by isolates recovered under both experimental conditions and does not indicate that a single isolate originated from both conditions. Species-level labels are shown for isolates with >97% sequence identity and phylogenetic clustering with the corresponding reference taxon and should be interpreted as tentative taxonomic assignments; otherwise, isolates are designated at the genus level. Bootstrap support values are shown on internal nodes and the scale bar indicates nucleotide substitutions per site. **(B)** Culturable bacterial abundance recovered on Luria–Bertani agar (LB), Nutrient Agar No. 1 (NA), Yeast Extract Agar (YEA), and Tryptic Soy Agar (TSA) in drought (orange) or control (blue) samples. Individual points represent independent biological replicates. Bars and error bars indicate mean ± SD of log_10_(CFU mL^-^¹ + 1). Asterisks indicate statistically significant differences between control and drought treatments after Benjamini–Hochberg adjustment across the four culture media (adjusted p < 0.05). Complete medium-specific statistical outputs are provided in **Supplementary Table S2**.

Culturable bacterial recovery on LB agar was higher under water limitation than under control conditions (Water-limitation − Control = 0.294 log_10_(CFU mL^-^¹ + 1); 95% CI, 0.174–0.415; adjusted P = 0.014; **Figure 6B**). No significant differences between treatments were detected on Nutrient Agar (adjusted P = 0.676), Yeast Extract Agar (adjusted P = 0.354), or Tryptic Soy Agar (adjusted P = 0.216). Thus, a treatment difference in culturable bacterial recovery was detected only on LB agar under the conditions tested.

Representative colonies were selected from each culture medium based on distinct morphological characteristics and identified by 16S rRNA gene sequencing. Phylogenetic analysis of the recovered isolates (**Figure 6A**) showed that isolates from control and water-limited plants were distributed across multiple phylogenetic lineages. Several closely related taxa were represented by isolates recovered under both treatments, whereas some lineages were represented only among isolates obtained from one treatment. Isolates affiliated with *Enterobacter* and *Novosphingobium* were recovered only from water-limited rhizosphere samples, whereas isolates affiliated with the *Xanthomonas* and *Bacillus* clades were recovered only from control rhizosphere samples. These observations describe watering-regime-specific recovery among the cultured isolates analysed and do not indicate taxon absence from the corresponding rhizosphere bacterial community or evidence of enrichment under the 40% FC regime.

## 4 Discussion

### 4.1 Water limitation alters seed nutritional and phytochemical composition with limited effects on dry reproductive biomass

Despite the absence of statistically detectable reductions in dry biomass, pod number and seed number (**Figures 1**, **2**), the observed changes in seed composition indicate that plant responses to water limitation extended beyond growth-related traits. The lower fresh biomass in the absence of a statistically detectable difference in dry biomass is compatible with differences in tissue water content, although plant water status was not directly measured. Similar patterns have been reported in legumes exposed to water limitation (Sehgal et al., 2018). Such responses are consistent with physiological adjustments that may help plants sustain reproductive development under stress, including osmotic adjustment, antioxidant defence, and hormonal regulation of water balance (Bagheri et al., 2023). The reduction in plant height (**Figure 2C**) further reflects the sensitivity of cell expansion processes to water-limiting conditions, which is among the earliest responses to water limitation in grain legumes (Sachdeva et al., 2022; Smith et al., 2019).

The absence of changes in chlorophyll content (**Figure 3C**) suggests that leaf greenness was not strongly affected under the imposed conditions, although SPAD values do not allow conclusions about photosynthetic performance. Water-limited plants produced similar aerial dry biomass with lower cumulative water input, resulting in a higher ratio of aerial dry biomass to cumulative water supplied (**Figures 3A, B**). Because this ratio combines biomass production and irrigation input, it is best interpreted as a descriptive normalisation rather than as a physiological measure of water-use efficiency. Transpiration, evaporation, and gas exchange were not directly measured in this experiment. These results nevertheless show that seed compositional changes occurred in the absence of a statistically detectable reduction in dry reproductive biomass, pod number, or seed number.

Endpoint-specific comparisons indicated lower seed N concentration, and, consequently, lower estimated crude protein content under 40% FC (**Figure 5A**, **Supplementary Table S1**), suggesting that water limitation may have affected N availability and/or allocation to developing seeds. However, because seed N did not remain supported after FDR adjustment within the seed nutritional endpoint family, this response should be interpreted as exploratory. Estimated crude protein was calculated directly from seed N using the conventional nitrogen-to protein conversion factor of 6.25. Consequently, the estimated crude-protein response reflects the observed change in seed N and does not constitute a separate measurement of seed protein. This distinction is important because nitrogen-to-protein conversion factors vary among crops and protein sources, and the general factor of 6.25 may overestimate true protein because not all seed N necessarily occurs as protein N (Mossé, 1990; Mariotti et al., 2008). In legumes, water limitation can restrict N nutrition through several non-exclusive mechanisms, including reduced N uptake and altered N remobilisation from vegetative reserves during reproductive development (Farooq et al., 2017). Similar reductions in seed N or estimated protein content under water limitation have been reported in several grain legumes (Sehgal et al., 2018; Machado et al., 2025), suggesting that this response may occur across species.

Among the minerals analysed, endpoint-specific comparisons indicated lower Zn concentration under lower water availability, while Mo showed a marginal decrease (**Figure 5B**). These responses did not remain supported after FDR adjustment (**Supplementary Table S1**) and should therefore be interpreted cautiously. They may nevertheless suggest that mineral acquisition and/or transport were sensitive to water limitation under the imposed conditions. Reduced nutrient mobility and mass flow due to low soil water availability can limit uptake and translocation within the plant (He and Dijkstra, 2014). These changes may be relevant for seed nutritional composition. However, the present data do not allow direct causal relationships between mineral status, N metabolism, and phytochemical accumulation to be established.

Water limitation also altered seed phytochemical composition (**Figure 4**), with increases in total saponin and flavonoids and a decrease in total phenolic content. These responses remained supported after FDR adjustment, indicating that the measured phytochemical traits were among the most consistent seed responses to water limitation in this experiment. The contrasting responses of total phenolics and flavonoids suggest that the watering regime affected these broad phytochemical indices differently. However, because the assays quantify global compound classes, rather than individual metabolites, the present data do not identify which specific phenolic, flavonoid, or saponin compounds were responsible for the observed differences. In *P. sativum* cv. Petit Provençal, Moisa et al. (2019) reported no statistically significant treatment differences in the phenolic compounds detected in seeds by targeted UHPLC analysis, including vanillic acid, syringic acid, and catechin. Direct comparison with the lower total phenolic index observed in the present study is limited because Moisa et al. (2019) quantified selected compounds chromatographically, whereas the present study measured a global colorimetric index, and the studies also differed in cultivar and watering regime. The decrease in the total phenolic index observed here also contrasts with the increase in total phenolic content commonly reported in drought-stressed chickpea and common bean (Herrera et al., 2019; Nisa et al., 2020). Collectively, these contrasting findings indicate that seed phenolic responses to water limitation are not uniform and may depend on species, genotype, treatment timing and intensity, seed developmental stage, growth conditions, and analytical methodology. The increase in total flavonoid content observed in the present experiment is compatible with the involvement of flavonoids in plant responses to water limitation, including antioxidant protection, as previously discussed in pea (Bagheri et al., 2023). Nevertheless, individual flavonoids were not identified, and no metabolic pathway or protective function was directly evaluated in the present study. Similarly, the marked increase in the total saponin index indicates a substantial change in this broad class of seed compounds, but the specific saponins involved and the mechanisms underlying this response remain unknown. These results should therefore be interpreted as watering-regime-associated differences in total phytochemical indices in P. sativum cv. Tom Thumb under the controlled conditions tested. In contrast, no statistically detectable difference was observed for phytic acid content (**Figure 4E**), despite reports of its accumulation under drought in other grain legumes (Machado et al., 2025), indicating that this response is not consistently triggered under water deficit.

The particularly strong increase in saponin content under water limitation conditions warrants further consideration. Saponins are secondary metabolites involved in plant defence, and their accumulation under abiotic stress has been reported in other legumes, including common bean (Herrera et al., 2019). Although often regarded as antinutritional compounds due to their potential to reduce mineral bioavailability, saponins have also been associated with beneficial health effects, including immunomodulatory and anticarcinogenic properties (Herrera et al., 2019). Therefore, the marked increase observed in this study (**Figure 4F**) may have implications for both seed nutritional quality and industrial processing. The co-occurrence of lower estimated protein content and increased saponin accumulation may be compatible with changes in carbon–nitrogen allocation under water limitation conditions, although this interpretation remains tentative because the N-related response did not remain supported after FDR adjustment. While such metabolic adjustments may contribute to stress responses, they can also affect seed nutritional quality.

The antioxidant responses observed through the ABTS and DPPH assays further reflect these compositional changes (**Figure 4**, **Supplementary Table S1**). ABTS antioxidant activity significantly increased under water limiting conditions, while DPPH showed a marginal decrease based on the endpoint-specific comparison. These divergent responses likely result from differences in assay sensitivity to distinct compound classes. The reduction in total phenolics may partly explain the lower DPPH response, whereas the concurrent rise in flavonoids and saponins may have contributed to the increased ABTS scavenging capacity. However, because individual phenolic and saponin compounds were not profiled, the contribution of specific metabolites remains to be clarified. In addition, detailed flowering and pod-set dates were not recorded; therefore, we cannot fully exclude the possibility that some compositional differences were partly influenced by seed developmental stage at harvest. Future studies including detailed phenological records and seed moisture measurements would help clarify the relationship between water limiting conditions, seed developmental stage, and seed composition.

Overall, water limitation had a clearer effect on seed phytochemical composition than on dry biomass accumulation and reproductive output. While no statistically detectable differences were observed in dry reproductive biomass, pod number, and seed number, lower water availability significantly altered several seed phytochemical traits, including saponins, total phenolics, total flavonoids, and antioxidant activity. Responses in seed N, estimated crude protein, Zn, and Mo suggest possible effects on seed nutritional composition, but these traits should be interpreted cautiously in light of the FDR sensitivity analysis. In the compact Tom Thumb genotype, these findings indicate that seed quality traits can respond to water limitation even when dry reproductive biomass is not detectably reduced. These results highlight the value of evaluating seed compositional traits alongside reproductive biomass, pod number and seed number, because limited treatment effects on these plant-level reproductive endpoints do not necessarily indicate unchanged seed composition. Studies across broader pea genetic diversity, different watering regimes, and growing environments will be needed before these traits can be considered in breeding or screening contexts.

### 4.2 Watering-regime-associated differences in culturable rhizosphere bacterial recovery

The culture-dependent analysis provided a complementary description of the culturable bacterial fraction recovered from control and water-limited pea rhizospheres. Because bacterial recovery depends on the ability of cells to grow and form colonies under the culture conditions used, this approach does not provide a quantitative description of the whole rhizosphere bacterial community (Vartoukian et al., 2010). The recovered isolates can nevertheless provide a basis for identifying culturable bacterial groups associated with the two watering regimes and for selecting isolates for subsequent functional characterisation.

Water limitation can alter soil water potential, nutrient mobility, and root exudation patterns and microbial survival in the rhizosphere (De Silva et al., 2025). These changes can influence which bacterial groups are recovered under contrasting water regimes. In the present study, reduced water availability had limited effects on culturable bacterial recovery across the four media tested. No significant differences were detected on Nutrient Agar, Yeast Extract Agar, or Tryptic Soy Agar, whereas recovery on LB agar was higher under the 40% field-capacity regime after Benjamini–Hochberg adjustment (**Figure 6B**, **Supplementary Table S2**). The response was therefore medium-specific rather than consistent across the culture conditions tested. Because the media provide different selective growth conditions, this may reflect differences in the bacterial groups preferentially recovered on each medium.

Beyond culturable abundance, water deficit may favour the persistence or recovery of bacterial groups with traits related to osmotic tolerance, metabolic flexibility, or interactions with plant-derived substrates (Adigoun et al., 2026; Hassani et al., 2018; Kost et al., 2024; Liu et al., 2025; Wang et al., 2026). Such responses are often taxon-specific and may contribute to differences in the culturable bacterial fraction recovered under contrasting watering regimes (Liu et al., 2025). Phylogenetic analysis showed that isolates recovered from rhizosphere samples associated with the 80% and 40% FC regimes were distributed across several bacterial lineages (**Figure 6A**). Several taxonomic groups were represented among isolates recovered under both watering regimes, whereas others were represented only among isolates recovered from one of the two regimes (**Figure 6A**). Isolates affiliated with *Enterobacter* and *Novosphingobium* were recovered only from rhizosphere samples collected under the 40% FC regime, whereas isolates affiliated with *Xanthomonas* and *Bacillus* clades were recovered only from samples collected under the 80% FC regime. These patterns describe treatment-specific recovery within the cultured isolate collection and do not demonstrate treatment-specific presence, enrichment, depletion, or absence in the rhizosphere community. In particular, failure to recover a taxon under one treatment cannot be interpreted as evidence that the taxon was absent from that rhizosphere. Taxonomic assignments were also interpreted conservatively because 16S rRNA gene sequencing does not always provide reliable species-level discrimination among closely related bacteria (Janda and Abbott, 2007).

Several genera presented among the recovered isolates, including *Enterobacter*, *Novosphingobium*, *Paenibacillus*, *Microbacterium*, and *Streptomyces*, contain strains previously associated with plant growth promotion or adaptation to abiotic stress conditions (Adigoun et al., 2026; Brambilla et al., 2022; Li et al., 2025). Reported traits within these genera include nutrient mobilisation, phytohormone production, siderophore synthesis, biofilm formation, and osmotic stress tolerance (**Supplementary Table S4**). Among the isolates recovered only from samples collected under the 40% FC regime, *Enterobacter* provides relevant literature context because strains from this genus have previously shown plant growth-promoting effects under salt and drought stress, including in rice and bermudagrass (Wei et al., 2022; Zhang et al., 2024). Members of *Novosphingobium* and *Microbacterium* have likewise been associated with plant growth promotion and persistence under stressful soil conditions, including reported roles in IAA production, biofilm formation, and C cycling (Canavera et al., 2026; Corretto et al., 2020). These reports provide biological context for the taxa recovered in the present study, but do not demonstrate that the isolates identified here expressed these functions under the experimental conditions used.

Overall, the culture-dependent approach recovered partly different bacterial isolate sets under the two watering regimes, while a treatment difference in culturable abundance was detected only on LB agar. Because the study relied on culture-dependent isolation, morphotype-based colony selection, and 16S rRNA-based gene identification of selected isolates, these findings should be interpreted as differences in the culturable fraction recovered under the experimental conditions rather than as quantitative changes in rhizosphere bacterial community composition. Because an unplanted substrate control was not included, the culture-dependent data do not allow us to distinguish bacteria already present in the commercial substrate from those preferentially associated with the pea rhizosphere. Future studies combining culture-independent microbiome profiling and functional characterisation of selected isolates will be needed to determine whether these bacteria have functional relevance for pea responses to water limitation and seed quality.

## 5 Conclusions

Water limitation at 40% FC altered the seed phytochemical profile of *P. sativum* cv. Tom Thumb under controlled environment-conditions, despite having limited effects on dry biomass, pod number, and seed number. The strongest seed responses were observed for saponins, flavonoids, total phenolics, and antioxidant activity. Endpoint-specific comparisons also indicated lower seed N and Zn concentrations, while Mo showed a marginal decrease. None of the seed nutritional or mineral responses remained supported after FDR adjustment and should therefore be considered exploratory.

At the rhizosphere level, reduced water availability was associated with differences in the culturable bacterial isolates recovered under the two watering regimes, while treatment effects on culturable bacterial abundance were detected only on LB agar. These findings indicate differences in the culturable bacterial fraction recovered under water-limited conditions, but the present data do not establish causal relationships between bacterial isolates and seed nutritional responses.

Overall, this study shows that seed compositional traits can respond to water limitation even when dry reproductive biomass is not detectably reduced. Future work integrating seed quality traits, culture-independent microbiome profiling, and functional analyses of selected isolates will be needed to clarify the responses of pea plants and their rhizosphere bacteria to reduced water availability.

## Data Availability

The datasets presented in this study can be found in online repositories. The names of the repository/repositories and accession number(s) can be found below: https://doi.org/10.5281/zenodo.22302087, 16S sequences for soil isolated bacteria.
