# Supplementary material for: Water limitation alters pea seed compositional traits and the culturable bacterial fraction recovered from the rhizosphere
Source: Front Plant Sci. 2026 Oct 12;17:1932989. doi: 10.3389/fpls.2026.1932989 (PMC13698824; doi:10.3389/fpls.2026.1932989)
Supplement: Supplementary file 1 [file Supplementaryfile1.docx]

**Figure S1.** Shoot fresh weight (A), pod fresh weight (B), seed fresh weight (C), and total aerial fresh biomass (D) of Pisum sativum cv. Tom Thumb plants subjected to well-watered control conditions (80% FC) or water-limited conditions (40% FC). Individual points represent independent biological replicates. Bars and error bars indicate mean ± SD. Asterisks indicate statistically significant differences based on the endpoint-specific statistical test described in Section 2.6. Endpoint-specific sample sizes, exact p-values, FDR-adjusted q-values, treatment effect estimates, 95% confidence intervals, and statistical tests are provided in Supplementary Table S1.

**Figure S2.** Representative *Pisum sativum* cv. Tom Thumb plants grown under well-watered control conditions (80% FC; left) and water-limited conditions (40% FC; right) under the controlled-environment conditions used in this study. The photograph was taken at 50 days after sowing and is provided as a representative visual record of plant phenotype.

**Table S1.** Endpoint-specific statistical outputs for quantitative traits analysed in Pisum sativum cv. Tom Thumb plants subjected to well-watered control conditions (80% FC) or water-limited conditions (40% FC). The table includes endpoint family, trait, statistical test, sample size per treatment, treatment effect estimate, confidence interval, exact p-value, FDR-adjusted q-value, and FDR discovery status.

| **Endpoint family** | **Figure reference** | **Trait**  **(unit)** | **Well-watered control conditions (80% FC)** | | **Water-limited conditions (40% FC).** | | **Satistical test** | **Effect estimate (40% FC – 80% FC)** | **Confidence interval** | **Exact *p*-value** | **FDR-adjusted q-value** | **FDR discovery** |
| --- | --- | --- | --- | --- | --- | --- | --- | --- | --- | --- | --- | --- |
|  |  |  | **mean ± SD** | **n** | **mean ± SD** | **n** |  |  |  |  |  |  |
| Growth/  physiology | Figure S1 | Shoot FW  (g) | 14.8±1.27 | 10 | 12.7±1.59 | 10 | Welch’s t-test | -2.130 | -3.484 to -0.776 | 0.0040 | 0.0074 | Yes |
| Growth/  physiology | Figure S1 | Pods FW  (g) | 7.77±0.66 | 10 | 6.82±0.75 | 10 | Welch’s t-test | -0.9500 | -1.615 to -0.285 | 0.0077 | 0.0094 | Yes |
| Growth/  physiology | Figure S1 | Seeds FW  (g) | 5.09±0.40 | 10 | 4.67±0.25 | 10 | Welch’s t-test | -0.4200 | -0.7350 to -0.105 | 0.0123 | 0.0129 | Yes |
| Growth/  physiology | Figure S1 | Total aerial biomass FW  (g) | 27.7±2.19 | 10 | 24.2±2.46 | 10 | Welch’s t-test | -3.500 | -5.693 to -1.307 | 0.0036 | 0.0074 | Yes |
| Growth/  physiology | Figure 2 | Pod number | 2.70±0.48 | 10 | 2.50±0.53 | 10 | Mann–Whitney test | 0.000 | -1.000 to 0.000 | 0.6499 | 0.3412 | No |
| Growth/  physiology | Figure 2 | Seed number | 10.4±1.17 | 10 | 9.70±0.82 | 10 | Mann–Whitney test | -1.000 | -2.000 to 0.000 | 0.2200 | 0.1348 | No |
| Growth/  physiology | Figure 2 | Plant height  (cm) | 20.9±1.63 | 10 | 18.6±1.29 | 10 | Welch’s t-test | -2.280 | -3.667 to -0.893 | 0.0029 | 0.0074 | Yes |
| Growth/  physiology | Figure 3 | Chlorophyll content  (SPAD values) | 32.5±4.23 | 10 | 36.8±7.16 | 10 | Welch’s t-test | 4.343 | -1.274 to 9.961 | 0.1199 | 0.0958 | No |
| Growth/  physiology | Figure 1 | Shoot DW  (g) | 0.87±0.06 | 8 | 0.80±0.09 | 8 | Welch’s t-test | -0.06935 | -0.1530 to 0.014 | 0.0962 | 0.0884 | No |
| Growth/  physiology | Figure 1 | Pods DW  (g) | 0.38±0.07 | 8 | 0.36±0.06 | 8 | Welch’s t-test | -0.02361 | -0.09501 to 0.048 | 0.4879 | 0.2759 | No |
| Growth/  physiology | Figure 1 | Seeds DW  (g) | 2.15±0.26 | 8 | 2.05±0.09 | 8 | Mann–Whitney test | -0.08610 | -0.2901 to 0.1019 | 0.1304 | 0.0958 | No |
| Growth/  physiology | Figure 3 | Total aerial biomass DW  (g) | 3.40±0.36 | 8 | 3.21±0.16 | 8 | Welch’s t-test | -0.1863 | -0.4982 to 0.1257 | 0.2121 | 0.1348 | No |
| Growth/  physiology | Figure 3 | Cumulative water supplied  (mL plant^-1^) | 2360±  113.9 | 10 | 1961±90.42 | 10 | Welch’s t-test | -399.1 | -496.1 to -302.1 | <0.0001 | 0.0007 | Yes |
| Growth/  physiology | Figure 3 | Aerial DW per cumulative water supplied  (mg mL^-1^) | 1.46±0.10 | 8 | 1.62±0.10 | 8 | Welch’s t-test | 0.1640 | 0.05284 to 0.2752 | 0.0069 | 0.0094 | Yes |
| Seed biochemical traits | Figure 4 | ABTS  (µmol TE g^-1^ DW) | 3.72±0.17 | 5 | 4.14±0.04 | 5 | Mann–Whitney test | 0.4066 | 0.1830 to 0.6080 | 0.0079 | 0.0055 | Yes |
| Seed biochemical traits | Figure 4 | DPPH  (µmol TE g^-1^ DW) | 2.33±0.06 | 5 | 2.17±0.13 | 5 | Welch’s t-test | -0.1602 | -0.3227 to 0.002 | 0.0523 | 0.0220 | Yes |
| Seed biochemical traits | Figure 4 | Total phenolics  (mg GAE g^-1^ DW) | 1.12±0.04 | 5 | 1.00±0.044 | 5 | Welch’s t-test | -0.1216 | -0.1858 to -0.0573 | 0.0024 | 0.0034 | Yes |
| Seed biochemical traits | Figure 4 | Total flavonoids  (mg CAE g^-1^ DW) | 0.50±0.03 | 5 | 0.57±0.02 | 5 | Welch’s t-test | 0.07095 | 0.03275 to 0.1091 | 0.0032 | 0.0034 | Yes |
| Seed biochemical traits | Figure 4 | Phytic acid  (mg g^-1^ DW) | 11.9±0.86 | 5 | 11.7±0.62 | 5 | Welch’s t-test | -0.1196 | -1.228 to 0.9886 | 0.8071 | 0.2825 | No |
| Seed biochemical traits | Figure 4 | Total Saponins  (mg DE g^-1^ DW) | 5.04±1.01 | 5 | 9.81±2.55 | 5 | Welch’s t-test | 4.670 | 1.625 to 7.715 | 0.0106 | 0.0056 | Yes |
| Seed nutritional traits | Figure 5 | N  (mg g^-1^ DW) | 31.81±1.16 | 5 | 29.4±1.03 | 5 | Welch’s t-test | -2.439 | -4.045 to -0.8335 | 0.0081 | 0.0765 | No |
| Seed nutritional traits | NA | Estimated crude protein  (mg g^-1^ DW) | 198.8±7.28 | 5 | 183.5±6.42 | 5 | Not tested independently; deterministic transformation of seed N | -15.25 | -25.29 to -5.22 | Same inference as seed N | Not included | Not included |
| Seed nutritional traits | Figure 5 | K  (mg g^-1^ DW) | 11.2±0.96 | 5 | 10.3±0.38 | 5 | Welch’s t-test | -0.9203 | -2.089 to 0.2487 | 0.0998 | 0.1886 | No |
| Seed nutritional traits | Figure 5 | P  (mg g^-1^ DW) | 4.37±0.42 | 5 | 4.11±0.28 | 5 | Welch’s t-test | -0.2630 | -0.7982 to 0.272 | 0.2824 | 0.3656 | No |
| Seed nutritional traits | Figure 5 | Ca  (mg g^-1^ DW) | 6.95±2.66 | 5 | 4.36±2.06 | 5 | Welch’s t-test | -2.583 | -6.094 to 0.9275 | 0.1270 | 0.2000 | No |
| Seed nutritional traits | Figure 5 | Mg  (mg g^-1^ DW) | 1.12±0.030 | 5 | 1.08±0.04 | 5 | Welch’s t-test | -0.04342 | -0.09372 to 0.00688 | 0.0812 | 0.1886 | No |
| Seed nutritional traits | Figure 5 | Fe  (µg g^-1^ DW) | 26.4±3.61 | 5 | 26.9±2.62 | 5 | Welch’s t-test | 0.5836 | -4.095 to 5.263 | 0.7780 | 0.8169 | No |
| Seed nutritional traits | Figure 5 | Zn  (µg g^-1^ DW) | 40.5±1.39 | 5 | 37.8±1.66 | 5 | Welch’s t-test | -2.690 | -4.928 to -0.451 | 0.0244 | 0.1153 | No |
| Seed nutritional traits | Figure 5 | Mo  (µg g^-1^ DW) | 4.70±1.05 | 5 | 3.37±0.71 | 5 | Welch’s t-test | -1.329 | -2.667 to 0.0084 | 0.0511 | 0.1610 | No |
| Seed nutritional traits | Figure 5 | Mn  (µg g^-1^ DW) | 11.3±0.86 | 5 | 10.40±0.94 | 5 | Mann–Whitney test | -1.286 | -2.095 to 0.7212 | 0.3095 | 0.3656 | No |

**Table note**: Group values are presented as mean ± SD. Effect estimates are expressed as 40% FC (water-limited conditions) – 80% FC (control conditions). For variables analysed using Welch’s unpaired t-test, effect estimates correspond to mean differences, and confidence intervals represent the corresponding 95% confidence intervals. For variables analysed using the Mann–Whitney test, effect estimates correspond to Hodges–Lehmann differences, with exact 95% confidence intervals reported as provided by Prism. Exact p-values refer to the endpoint-specific statistical tests. FDR-adjusted q-values were calculated separately within biologically defined endpoint families using the two-stage step-up method of Benjamini, Krieger and Yekutieli, with Q set to 5%. “FDR discovery” indicates whether an endpoint met the corresponding FDR criterion. Estimated crude protein was calculated directly from seed N using a nitrogen-to-protein conversion factor of 6.25. Its group means, SDs, effect estimate, and 95% confidence interval are deterministic transformations of the corresponding seed N values. Estimated crude protein was not tested or included in the FDR sensitivity analysis as an independent endpoint.

**Table S2.** Medium-specific statistical outputs for culturable bacterial recovery in the rhizosphere of *Pisum sativum* cv. Tom Thumb plants subjected to well-watered control conditions (80% FC) or water-limited conditions (40% FC). The table includes culture medium, sample size per treatment, treatment means ± SD, treatment effect estimate, 95% confidence interval, Welch’s t-statistic, degrees of freedom, exact p-value, and Benjamini–Hochberg-adjusted p-value.

| **Culture medium** | **Well-watered control conditions (80% FC)** | | **Water-limited conditions (40% FC).** | | **Effect estimate (40% FC – 80% FC)** | **Confidence interval** | **Welch's *t*** | **df** | **Exact *p-value*** | **BH-adjusted *p*** |
| --- | --- | --- | --- | --- | --- | --- | --- | --- | --- | --- |
|  | **mean ± SD** | ***n*** | **mean ± SD** | ***n*** |  |  |  |  |  |  |
| LB Agar | 2.493 ± 0.015 | 3 | 2.787 ± 0.041 | 3 | 0.294 | 0.174–0.415 | 7.227 | 3.447 | 0.003 | 0.014 |
| Nutrient Agar | 3.409 ± 0.093 | 3 | 3.354 ± 0.117 | 3 | −0.054 | −0.396–0.287 | -0.452 | 3.797 | 0.676 | 0.676 |
| Yeast Extract Agar | 2.903 ± 0.119 | 3 | 2.709 ± 0.061 | 3 | −0.194 | −0.626–0.238 | -1.325 | 3.475 | 0.265 | 0.354 |
| Tryptic Soy Agar (TSA) | 1.981 ± 0.068 | 3 | 2.306 ± 0.155 | 3 | 0.325 | −0.165–0.815 | 2.657 | 2.166 | 0.108 | 0.216 |

**Table note**: Culturable bacterial abundance was expressed as log₁₀(CFU mL⁻¹ + 1). Technical replicate plates were averaged within each independent biological replicate before statistical analysis. Group values are presented as mean ± SD. Effect estimates are expressed as 40% FC (water-limited conditions) – 80% FC (control conditions) and correspond to differences between treatment means, with the associated 95% confidence intervals. Well-watered control conditions (80% FC) and water-limited conditions (40% FC) were compared separately within each culture medium using Welch’s two-sample t-test. Exact p-values refer to the medium-specific tests. P-values were adjusted across the four culture media using the Benjamini–Hochberg procedure, with adjusted p < 0.05 considered statistically significant.

**Table S3.** Identification and metadata of culturable bacterial isolates recovered from pea rhizosphere samples under well-watered control conditions (80% FC) or water-limited conditions (40% FC). For each isolate, the table reports the closest BLASTn hit, sequence identity, query coverage, E-value, GenBank accession used as the tree proxy, rhizosphere condition, isolation dilution, culture medium, and experimental colony morphology. Taxonomic groups represented in both watering regimes are indicated as Shared. Isolates excluded from the phylogenetic analysis are reported separately together with the corresponding exclusion criteria.

| **Isolate (ISO) ID** | **Closest BLAST Hit** | **Identity (%)** | **Coverage (%)** | **E-value** | **GenBank Accession (Tree Proxy)** | **Rhizosphere Condition** | **Isolation Dilution** | **Isolation Medium** | **Experimental Morphology (Colony Type)** | **Morphology Criteria** |  |
| --- | --- | --- | --- | --- | --- | --- | --- | --- | --- | --- | --- |
| **1** | *Flavobacterium* sp. CJ75 | 95.71 | 98 | 0 | CP119766.1 (Proxy: ON697206.1) | Shared | 10⁻² | Nutrient Agar | **J** (Yellow/orange, round, translucent) | Circular, convex, smooth, yellow-pigmented, entire margin |  |
| **2** | *Chryseobacterium* sp. CS-F4 | 95.57 | 99 | 0 | MN022538.1 | Water-limited conditions s | 10⁻¹ | Nutrient Agar | **J** (Yellow/orange, round, translucent) | Circular, smooth, glistening, yellow to orange pigmented, entire margin |  |
| **3** | *Novosphingobium resinovorum* JCM 12332 | 95.61 | 90 | 0 | LC507452.1 | Water-limited conditions | 10⁻³ | Yeast Extract Agar | **E** (Small, white, irregular flocs) | Circular, convex, smooth, yellowish/pinkish, translucent |  |
| **4** | *Microbacterium natoriense* strain 788 | 95.75 | 91 | 0 | MT367754.1 | Shared | 10⁻² | Tryptic Soy Agar | **D** (Yellowish, round, medium-sized) | Circular, convex, smooth, yellow to orange-yellow pigmented, entire margin |  |
| **6** | *Streptomyces spiralis* S2Cb-1.13 | 100 | 91 | 1×10⁻⁸⁰ | KT992135.1 | Control | 10⁻¹ | Yeast Extract Agar | **B** (Small white/greyish, faded frame) | Porous, filamentous, leathery, often with aerial mycelium |  |
| **7** | *Streptomyces thermocarboxydus* EGI124 | 100 | 92 | 1×10⁻⁸⁰ | MN704433.1 | Control | 10⁻³ | Yeast Extract Agar | **B** (Small white/greyish, faded frame) | Porous, filamentous, leathery, often with aerial mycelium |  |
| **9** | *Luteimicrobium* sp. G6 | 97 | 99 | 0 | MT993404.1 | Shared | 10⁻² | Tryptic Soy Agar | **D** (Yellowish, round, medium-sized) | Circular, convex, yellow-pigmented, smooth |  |
| **11** | *Bacillus thuringiensis* GCU-SI-02 | 97.96 | 81 | 0 | MT126823.1 | Control | 10⁻¹ | LB Agar | **M** (Milky smear/irregular) | Large, irregular or circular, flat to convex, rough/matte or glossy, opaque, spreading |  |
| **15** | *Microbacterium hydrocarbonoxydans* Y20 | 97.85 | 100 | 0 | JN084159.1 | Control | 10⁻³ | Tryptic Soy Agar | **D** (Yellowish, round, medium-sized) | Circular, convex, smooth, yellow to orange-yellow pigmented, entire margin |  |
| **16** | *Cellulomonas massiliensis* AFS033555 | 96.85 | 80 | 0 | OP986550.1 | Water-limited conditions | 10⁻² | Tryptic Soy Agar | **A** (White round, small) | Short rods, circular, convex, yellow/cream, entire |  |
| **17** | *Chryseobacterium* sp. VV8 | 99.2 | 87 | 0 | MG725656.1 | Control | 10⁻¹ | Nutrient Agar | **J** (Yellow/orange, round, translucent) | Circular, smooth, glistening, yellow to orange pigmented, entire margin |  |
| **18** | *Bacillus* sp. L244 | 98.13 | 88 | 0 | AM913937.1 | Control | 10⁻³ | LB Agar | **M** (Milky smear/irregular) | Large, irregular or circular, flat to convex, rough/matte or glossy, opaque, spreading |  |
| **20** | *Rhodanobacter ginsenosidimutans* | 97.92 | 99 | 0 | AB682427.1 | Shared | 10⁻² | Tryptic Soy Agar | **C** (Coma/elliptical shape) | Circular, smooth, flat to convex, yellow/greenish-yellow |  |
| **22** | *Luteimicrobium* sp. RB22 | 97.44 | 85 | 0 | KY558708.2 | Control | 10⁻¹ | Tryptic Soy Agar | **D** (Yellowish, round, medium-sized) | Circular, convex, yellow-pigmented, smooth |  |
| **23** | *Shinella fusca* E3-12 | 97.47 | 94 | 0 | OQ753139.1 | Water-limited conditions | 10⁻³ | Yeast Extract Agar | **P** (Pinkish bacteria) | Circular, convex, pink to beige, translucent |  |
| **31** | *Rhodanobacter* sp. A11 | 96.42 | 99 | 0 | MH688757.1 | Water-limited conditions | 10⁻² | Tryptic Soy Agar | **C** (Coma/elliptical shape) | Circular, smooth, flat to convex, yellow/greenish-yellow |  |
| **33** | *Microbacterium* sp. RJ-12-2 | 97.7 | 79 | 0 | MN176476.1 | Control | 10⁻¹ | Tryptic Soy Agar | **D** (Yellowish, round, medium-sized) | Circular, convex, smooth, yellow to orange-yellow pigmented, entire margin |  |
| **34** | *Microbacterium hydrocarbonoxydans* HM58-2 | 95.14 | 84 | 0 | LC005742.1 | Control | 10⁻³ | Tryptic Soy Agar | **D** (Yellowish, round, medium-sized) | Circular, convex, smooth, yellow to orange-yellow pigmented, entire margin |  |
| **35** | *Microbacterium* sp. KUDC0406 | 95.76 | 94 | 0 | CP091138.1 (Proxy: X77441.1) | Water-limited conditions | 10⁻² | Tryptic Soy Agar | **D** (Yellowish, round, medium-sized) | Circular, convex, smooth, yellow to orange-yellow pigmented, entire margin |  |
| **36** | *Microbacterium lacticum* D21 | 97.72 | 87 | 0 | OL614736.1 | Water-limited conditions | 10⁻¹ | Tryptic Soy Agar | **D** (Yellowish, round, medium-sized) | Circular, convex, smooth, yellow to orange-yellow pigmented, entire margin |  |
| **37** | *Streptomyces* sp. PU-MM82 | 100 | 91 | 2×10⁻⁷⁷ | MK907517.1 | Water-limited conditions | 10⁻³ | Yeast Extract Agar | **B** (Small white/greyish, faded frame) | Porous, filamentous, leathery, often with aerial mycelium |  |
| **38** | *Paenibacillus polymyxa* MJ4 | 97.93 | 78 | 0 | MT367718.1 | Water-limited conditions | 10⁻² | LB Agar | **M** (Milky smear/irregular) | Circular to irregular, creamy to yellowish, smooth to rough |  |
| **39** | *Microbacterium foliorum* DG-42 | 98.22 | 96 | 0 | OR098490.1 | Water-limited conditions | 10⁻¹ | Tryptic Soy Agar | **D** (Yellowish, round, medium-sized) | Circular, convex, smooth, yellow to orange-yellow pigmented, entire margin |  |
| **43** | *Microbacterium phyllosphaerae* SS108 | 95.59 | 97 | 0 | HG941981.1 | Water-limited conditions | 10⁻³ | Tryptic Soy Agar | **D** (Yellowish, round, medium-sized) | Circular, convex, smooth, yellow to orange-yellow pigmented, entire margin |  |
| **44** | *Novosphingobium resinovorum* EN14ZR1 | 97.16 | 79 | 0 | KY486807.1 | Water-limited conditions | 10⁻² | Yeast Extract Agar | **E** (Small, white, irregular flocs) | Circular, convex, smooth, yellowish/pinkish, translucent |  |
| **45** | *Enterobacter ludwigii* Xuyi_356_1 | 96.87 | 94 | 0 | MN310507.1 | Water-limited conditions | 10⁻¹ | LB Agar | **N** (White with visible ring inside) | Circular, convex, smooth, creamy-white, translucent, entire |  |
| **47** | *Chryseobacterium* sp. 549 | 96.95 | 95 | 0 | EF565935.1 | Water-limited conditions | 10⁻³ | Nutrient Agar | **J** (Yellow/orange, round, translucent) | Circular, smooth, glistening, yellow to orange pigmented, entire margin |  |
| **48** | *Pseudomonas koreensis* 96C1 | 97.58 | 84 | 0 | MT012074.1 | Water-limited conditions | 10⁻² | LB Agar | **C** (Coma/elliptical shape) | Circular, smooth, flat to convex, greenish-yellow fluorescent or creamy, opaque |  |
| **49** | *Microbacterium* sp. Tm-B5 | 97.13 | 99 | 0 | MT533987.1 | Water-limited conditions | 10⁻¹ | Tryptic Soy Agar | **D** (Yellowish, round, medium-sized) | Circular, convex, smooth, yellow to orange-yellow pigmented, entire margin |  |
| **53** | *Microbacterium saccharophilum* SS1 | 96.6 | 96 | 0 | OK090551.1 | Water-limited conditions | 10⁻³ | Tryptic Soy Agar | **D** (Yellowish, round, medium-sized) | Circular, convex, smooth, yellow to orange-yellow pigmented, entire margin |  |
| **56** | *Flavobacterium* sp. PYRL32 | 96.92 | 98 | 0 | MF458891.1 | Control | 10⁻² | Nutrient Agar | **J** (Yellow/orange, round, translucent) | Circular, convex, smooth, yellow-pigmented, entire margin |  |
| **57** | *Flavobacterium* sp. Hc031 | 98.17 | 89 | 0 | KY465525.1 | Control | 10⁻¹ | Nutrient Agar | **J** (Yellow/orange, round, translucent) | Circular, convex, smooth, yellow-pigmented, entire margin |  |
| **59** | *Paenibacillus cineris* cu1-7 | 97.14 | 98 | 0 | MT373481.1 | Control | 10⁻³ | LB Agar | **M** (Milky smear/irregular) | Circular to irregular, creamy to yellowish, smooth to rough |  |
| **60** | *Pseudomonas* sp. zw47 | 98.13 | 90 | 0 | MH337959.1 | Control | 10⁻² | LB Agar | **C** (Coma/elliptical shape) | Circular, smooth, flat to convex, greenish-yellow fluorescent or creamy, opaque |  |
| **61** | *Castellaniella denitrificans* RS-MT7 | 96.22 | 100 | 0 | KF371657.1 | Control | 10⁻¹ | Tryptic Soy Agar | **P** (Pinkish bacteria) | Circular, convex, smooth, cream to pale pink |  |
| **63** | *Xanthomonas maliensis* BT5 | 95.98 | 80 | 4×10⁻⁷¹ | MK092984.1 | Control | 10⁻³ | Nutrient Agar | **G** (Yellowish circles, big, milky) | Circular, convex, smooth, viscous/mucoid, deep yellow pigmented |  |
| **65** | *Xanthomonas maliensis* B6B | 95.86 | 100 | 0 | MW142058.1 | Control | 10⁻² | Nutrient Agar | **G** (Yellowish circles, big, milky) | Circular, convex, smooth, viscous/mucoid, deep yellow pigmented |  |
| **70** | *Pseudomonas corrugata* Pc.Sl.2011 | 96.64 | 99 | 0 | KC812729.1 | Shared | 10⁻¹ | LB Agar | **C** (Coma/elliptical shape) | Circular, smooth, flat to convex, greenish-yellow fluorescent or creamy, opaque |  |
| **73** | *Paenibacillus polymyxa* Sb3-1 | 98.23 | 88 | 0 | CP010268.1 (Proxy: AJ320493.1) | Shared | 10⁻³ | LB Agar | **M** (Milky smear/irregular) | Circular to irregular, creamy to yellowish, smooth to rough |  |
| **74** | *Enterobacter* sp. TC106 | 95.62 | 88 | 0 | MK459474.1 | Water-limited conditions | 10⁻² | LB Agar | **N** (White with visible ring inside) | Circular, convex, smooth, creamy-white, translucent, entire |  |
| **75** | *Streptomyces* sp. EGI63005 | 98.3 | 88 | 0 | MK045628.1 | Control | 10⁻¹ | Yeast Extract Agar | **B** (Small white/greyish, faded frame) | Porous, filamentous, leathery, often with aerial mycelium |  |
| **77** | *Microbacterium natoriense* P1_23 | 98.45 | 86 | 0 | LT838020.1 | Control | 10⁻³ | Tryptic Soy Agar | **D** (Yellowish, round, medium-sized) | Circular, convex, smooth, yellow to orange-yellow pigmented, entire margin |  |
| **78** | *Flavobacterium* sp. H7R.22 | 96.38 | 90 | 0 | MK138867.1 | Water-limited conditions | 10⁻² | Nutrient Agar | **J** (Yellow/orange, round, translucent) | Circular, convex, smooth, yellow-pigmented, entire margin |  |
| **81** | *Sphingobacterium siyangense* PDNC006 | 97.89 | 99 | 0 | CP070350.1 (Proxy: EU046272.1) | Water-limited conditions | 10⁻¹ | Tryptic Soy Agar | **E** (Small, white, irregular flocs) | Circular, smooth, glistening, cream to yellowish |  |
| **82** | *Enterobacter ludwigii* ST10 | 96.44 | 97 | 0 | MK894868.1 | Water-limited conditions | 10⁻³ | LB Agar | **N** (White with visible ring inside) | Circular, convex, smooth, creamy-white, translucent, entire |  |
| **84** | *Microbacterium* sp. ZKA43 | 97.64 | 91 | 0 | MT683214.1 | Water-limited conditions | 10⁻² | Tryptic Soy Agar | **D** (Yellowish, round, medium-sized) | Circular, convex, smooth, yellow to orange-yellow pigmented, entire margin |  |
| **Isolate (ISO) ID** | **Closest BLAST Hit** | **Identity (%)** | **Coverage (%)** | **E-value** | **GenBank Accession** | **Isolation Condition** | **Isolation Medium** | **Isolation Dilution** | **Experimental Morphology (Colony Type)** | **Morphology (Reference)** | **Note (Exclusion Reason)** |
| **12** | *Novosphingobium* sp. YZX-2 | 83.74 | 58 | 8×10⁻¹⁵⁹ | MT270803.1 | Control | Yeast Extract Agar | 10⁻¹ | **E** (Small, white, irregular flocs) | Circular, convex, smooth, yellowish/pinkish, translucent | Low identity + coverage. Identity <95% and coverage <70% |
| **13** | *Bosea* sp. Alpha-65 | 84.12 | 66 | 1×10⁻³⁶ | MH686115.1 | Control | Yeast Extract Agar | 10⁻² | **P** (Pinkish bacteria) | Circular, convex, smooth, beige to pale pink | Low identity + coverage. Identity <95% and coverage <70% |
| **21** | Uncultured gamma proteobacterium clone g13 | 70.31 | 44 | 2×10⁻¹⁰ | EU979022.1 | Control | Tryptic Soy Agar | 10⁻³ | **A** (White round, small) | Circular, smooth, translucent, cream-colored | Identity <95%, coverage <70%, uncultured sequence |
| **25** | *Lysinibacillus massiliensis* NAE 2-2 | 97.62 | 17 | 3×10⁻⁹⁴ | KC603539.1 | Control | LB Agar | 10⁻¹ | **M** (Milky smear/irregular) | Circular, irregular, opaque, rough or smooth, creamy-white | Coverage <70% |
| **28** | *Lactobacillus fermentum* 13-3-1-2 | 94.12 | 5 | 3×10⁻⁹ | EU287483.1 | Control | Yeast Extract Agar | 10⁻² | **A** (White round, small) | Circular, convex, smooth, white to creamy, entire margin | Identity <95% and coverage <70% |
| **46** | *Rhodococcus qingshengii* RL1 | 91.76 | 90 | 9×10⁻¹⁰⁴ | CP042917.1 | Control | Tryptic Soy Agar | 10⁻¹ | **S** (Long smear, orange) | Circular, smooth to rugose, convex, bright orange-red or pinkish | Identity <95% |
| **54** | *Pseudoalteromonas* sp. V2-173 | 97.22 | 12 | 7×10⁻⁵ | JN106693.1 | Control | Tryptic Soy Agar | 10⁻³ | **I** (Big, circular, faded) | Circular, smooth, translucent, beige to yellowish | Coverage <70%+ weak E-value |
| **66** | Uncultured Flavobacterium clone 7849 | 84.44 | 85 | 2×10⁻⁸¹ | KF508151.1 | Control | Nutrient Agar | 10⁻² | **J** (Yellow/orange, round, translucent) | Circular, convex, smooth, yellow-pigmented, entire margin | Low identity + coverage. Identity <95% and coverage <70% |
| **69** | *Cytobacillus firmus* O1 | 94.36 | 26 | 3×10⁻⁷⁷ | OL519522.1 | Control | LB Agar | 10⁻¹ | **M** (Milky smear/irregular) | Large, irregular, flat to convex, rough, opaque | Low identity + coverage. Identity <95% and coverage <70% |
| **76** | Uncultured prokaryote clone 17469 | 94 | 3 | 1×10⁻⁸ | KT055222.1 | Control | Tryptic Soy Agar | 10⁻³ | **A** (White round, small) | Circular, small, punctiform, cream-colored | Identity <95%, coverage <70%, uncultured sequence |
| **83** | Uncultured bacterium clone A814 | 81.15 | 11 | 6×10⁻¹⁶ | MW878600.1 | Control | Tryptic Soy Agar | 10⁻² | **A** (White round, small) | Circular, smooth, translucent, cream-colored | Identity <95%, coverage <70%, uncultured sequence |

**Table note**: Taxonomic assignments were based on percentage identity, query coverage, E-value, and consistency among the top BLASTn hits. Species names reported under “Closest BLASTn hit” indicate the closest database match and should not be interpreted as definitive species-level identifications. Taxonomic assignments used in the manuscript were conservative and limited to the lowest confidently supported taxonomic rank, particularly for isolates with lower sequence identity or query coverage. Experimental colony morphology was recorded to document the visually distinct morphotypes used for colony selection and was not used as taxonomic evidence. Shared indicates taxonomic groups represented by isolates recovered under samples under well-watered control conditions (80% FC) or water-limited conditions (40% FC). 27 isolates were excluded before analysis due to poor sequencing quality or mixed chromatograms indicative of co-isolation and are not included in this table.

**Table S4.** Literature-based functional traits associated with bacterial taxa recovered from the rhizosphere of *Pisum sativum* cv. Tom Thumb subjected to well-watered control conditions (80% FC) or water-limited conditions (40% FC). The table summarizes previously reported plant-associated characteristics of the recovered bacterial taxa. The information is provided as context for future functional screening and should not be interpreted as experimentally validated functions of the isolates obtained in this study.

| **Taxon** | **Osmotic tolerance** | **Hormone modulation** | **Biofilm/colonization** | **Nutrient cycling** | **Antioxidant / ROS** | **Notes** | **References** |
| --- | --- | --- | --- | --- | --- | --- | --- |
| *Enterobacter ludwigii* | Reported in some strains | IAA / ACC (strain-dependent) | Yes | N cycling | Reported | Drought-enriched; known PGPR group | [1], [2] |
| *Novosphingobium spp.* | Reported | Limited evidence | Strong biofilms | Aromatic C degradation | Limited data | Stress-tolerant lineage | [3], [4] |
| *Rhodococcus qingshengii* | Reported | Limited evidence | Yes | C/xenobiotic cycling | Reported | Actinobacteria with stress tolerance | [5], [6] |
| *Rhodanobacter spp.* | Reported | Poorly characterized | Limited data | Denitrification | Limited data | Associated with N-cycle shifts | [7] |
| *Pseudomonas koreensis* | Yes (well documented) | IAA / siderophores | Strong colonizer | Yes | Yes | Well-established PGPR | [8], [9] |
| *Paenibacillus polymyxa* | Yes | IAA / ACC | Yes | N/P cycling | Yes | Classic PGPR species | [10] |
| *Cellulomonas massiliensis* | Reported | Limited evidence | Limited data | Cellulose degradation | Limited data | Decomposer; possible stress adaptation | [11] |
| *Microbacterium spp.* | Strain-dependent | IAA (some strains) | Yes | C cycling | Reported | Mixed response; common plant-associated genus | [12] |
| *Bacillus spp.* | Yes (many strains) | IAA / ABA (strain-dependent) | Strong (biofilms, spores) | Yes | Yes | Broad PGPR group | [13] |
| *Chryseobacterium spp.* | Limited data | Limited data | Yes | Proteolytic activity | Limited data | Increasing evidence in plant systems | [14] |
| *Streptomyces spp.* | Yes (many strains) | Some strains | Yes | C cycling | Yes | Often plant-beneficial despite control enrichment | [15] |
| *Flavobacterium spp.* | Variable | Limited evidence | Yes | Organic matter degradation | Limited data | Common rhizosphere colonizers | [16] |
| *Luteimicrobium spp.* | Poorly characterized | Unknown | Unknown | Likely C cycling | Unknown | Limited literature | [17] |

[1]	M. Shoebitz, C. M. Ribaudo, M. A. Pardo, M. L. Cantore, L. Ciampi, and J. A. Curá, “Plant growth promoting properties of a strain of *Enterobacter ludwigii* isolated from *Lolium perenne* rhizosphere,” *Soil Biol. Biochem.*, vol. 41, no. 9, pp. 1768–1774, Sep. 2009, doi: 10.1016/J.SOILBIO.2007.12.031.

[2]	J. Zhang *et al.*, “*Enterobacter ludwigii* b3 in the rhizosphere of wild rice assists cultivated rice in mitigating drought stress by direct and indirect methods,” *Biochem. Biophys. Res. Commun.*, vol. 735, p. 150489, Nov. 2024, doi: 10.1016/J.BBRC.2024.150489.

[3]	R. Kumar *et al.*, “Comparative Genomic Analysis Reveals Habitat-Specific Genes and Regulatory Hubs within the Genus Novosphingobium,” *mSystems*, vol. 2, no. 3, May 2017, doi: 10.1128/MSYSTEMS.00020-17.

[4]	F. Terán, V. Vives-Peris, A. Gómez-Cadenas, and R. M. Pérez-Clemente, “Enhancing citrus resilience to high temperature and intense light stress with *Pseudomonas putida* and *Novosphingobium sp*.,” *Environ. Exp. Bot.*, vol. 235, p. 106167, Jul. 2025, doi: 10.1016/J.ENVEXPBOT.2025.106167.

[5]	L. Iminova *et al.*, “Physiological and biochemical characterization and genome analysis of *Rhodococcus qingshengii* strain 7B capable of crude oil degradation and plant stimulation,” *Biotechnology Reports*, vol. 35, p. e00741, Sep. 2022, doi: 10.1016/J.BTRE.2022.E00741.

[6]	T. Kuhl, S. P. Chowdhury, J. Uhl, and M. Rothballer, “Genome-Based Characterization of Plant-Associated *Rhodococcus qingshengii* RL1 Reveals Stress Tolerance and Plant-Microbe Interaction Traits,” *Front. Microbiol.*, vol. 12, Aug. 2021, doi: 10.3389/FMICB.2021.708605.

[7]	O. Prakash, S. J. Green, P. Singh, P. Jasrotia, and J. E. Kostka, “Stress-related ecophysiology of members of the genus *Rhodanobacter* isolated from a mixed waste contaminated subsurface,” *Frontiers of Environmental Science & Engineering 2020 15:2*, vol. 15, no. 2, pp. 23-, Sep. 2020, doi: 10.1007/S11783-020-1315-0.

[8]	Q. Guo *et al.*, “*Pseudomonas koreensis* promotes tomato growth and shows potential to induce stress tolerance via auxin and polyphenol‐related pathways,” *Plant and Soil 2021 462:1*, vol. 462, no. 1, pp. 141–158, Feb. 2021, doi: 10.1007/S11104-021-04837-9.

[9]	J. N. Kalleku *et al.*, “Halotolerant *Pseudomonas koreensis* S4T10 mitigate salt and drought stress in *Arabidopsis thaliana*,” *Physiol. Plant.*, vol. 176, no. 2, Mar. 2024, doi: 10.1111/PPL.14258.

[10]	Y. Qin *et al.*, “Plant Growth-Promoting Rhizobacteria *Paenibacillus polymyxa* HL14-3 Inoculation Enhances Drought Tolerance in Cucumber by Triggering Abscisic Acid-Mediated Stomatal Closure,” *J. Agric. Food Chem.*, vol. 73, no. 1, pp. 260–272, Jan. 2025, doi: 10.1021/ACS.JAFC.4C09421.

[11]	L. Wu *et al.*, “Identification, characteristics and rice growth promotion of a highly efficient cellulolytic bacterial strain, *Cellulomonas iranensis* ZJW-6, isolated from paddy soil in central China,” *Front. Microbiol.*, vol. 14, p. 1152966, 2023, doi: 10.3389/FMICB.2023.1152966/FULL.

[12]	E. Corretto *et al.*, “Comparative Genomics of Microbacterium Species to Reveal Diversity, Potential for Secondary Metabolites and Heavy Metal Resistance,” *Front. Microbiol.*, vol. 11, p. 1869, Aug. 2020, doi: 10.3389/FMICB.2020.01869/FULL.

[13]	H. Etesami, B. R. Jeong, and B. R. Glick, “Potential use of *Bacillus spp.* as an effective biostimulant against abiotic stresses in crops—A review,” *Curr. Res. Biotechnol.*, vol. 5, p. 100128, Jan. 2023, doi: 10.1016/J.CRBIOT.2023.100128.

[14]	H. Wang *et al.*, “The regulatory mechanism of *Chryseobacterium sp.* resistance mediated by montmorillonite upon cadmium stress,” *Chemosphere*, vol. 240, Feb. 2020, doi: 10.1016/J.CHEMOSPHERE.2019.124851.

[15]	Z. Yang *et al.*, “*Streptomyces* alleviate abiotic stress in plant by producing pteridic acids,” *Nature Communications 2023 14:1*, vol. 14, no. 1, pp. 7398-, Nov. 2023, doi: 10.1038/s41467-023-43177-3.

[16]	H. Seo, J. H. Kim, S. M. Lee, and S. W. Lee, “The Plant-Associated Flavobacterium: A Hidden Helper for Improving Plant Health,” *Plant Pathol. J.*, vol. 40, no. 3, p. 251, Jun. 2024, doi: 10.5423/PPJ.RW.01.2024.0019.

[17]	M. Hamada, H. Yamamura, C. Komukai, T. Tamura, K. I. Suzuki, and M. Hayakawa, “*Luteimicrobium album sp*. nov., a novel actinobacterium isolated from a lichen collected in Japan, and emended description of the genus *Luteimicrobium*,” *J. Antibiot. (Tokyo).*, vol. 65, no. 8, pp. 427–431, Aug. 2012, doi: 10.1038/JA.2012.45.
